# Phyllosphere microbial community of cigar tobacco and its corresponding metabolites

**DOI:** 10.3389/fmicb.2022.1025881

**Published:** 2022-11-11

**Authors:** Tiantian Liu, Shiping Guo, Chongde Wu, Ruina Zhang, Qiu Zhong, Hongzhi Shi, Rongqing Zhou, Yanqing Qin, Yao Jin

**Affiliations:** ^1^College of Biomass Science and Engineering, Sichuan University, Chengdu, China; ^2^Key Laboratory of Leather Chemistry and Engineering, Ministry of Education, Sichuan University, Chengdu, China; ^3^R&D Department, Sichuan Provincial Branch of China National Tobacco Crop Tobacco Science Institute, Chengdu, China; ^4^R&D Department, Deyang Tobacco Company of Sichuan Province, Sichuan, Deyang, China; ^5^College of Tobacco Science, Henan Agricultural University, Zhengzhou, China

**Keywords:** cigar fermentation, microbial community, volatile metabolites, high throughput sequencing, GC–MS

## Abstract

Cigar is made of a typical fermented tobacco where the microbiota inhabits within an alkaline environment. Our current understanding on cigar fermentation is far from thorough. This work employed both high-throughput sequencing and chromatography-mass spectrometric technologies to provide new scientific reference for this specific fermented system. Typical cigar samples from different regions (the Caribbeans, South America, East Asia, and Southeast Asia) were investigated. The results show that *Firmicutes*, *Actinobacteria*, *Proteobacteria, Ascomycota*, and *Basidiomycota* were the predominant phyla in the cigar samples. Rather than the fungal community, it was the bacterial community structures that played vital roles to differentiate the cigar from different regions: *Staphylococcus* was the dominant genus in the Americas; *Bacillus* was the dominant genus in Southeast Asia; while in East Asia, there was no dominant genus. Such differences in community structure then affected the microflora metabolism. The correlation between microbiota and metabolites revealed that *Aspergillaceae*, *Cercospora*, and *Staphylococcus* were significantly correlated with sclareolide; *Bacillus* were positively associated with isophorone. *Alcaligenaceae* was significantly and positively correlated with L-nicotine and hexadecanoic acid, methyl ester.

**GRAPHICAL ABSTRACT:**
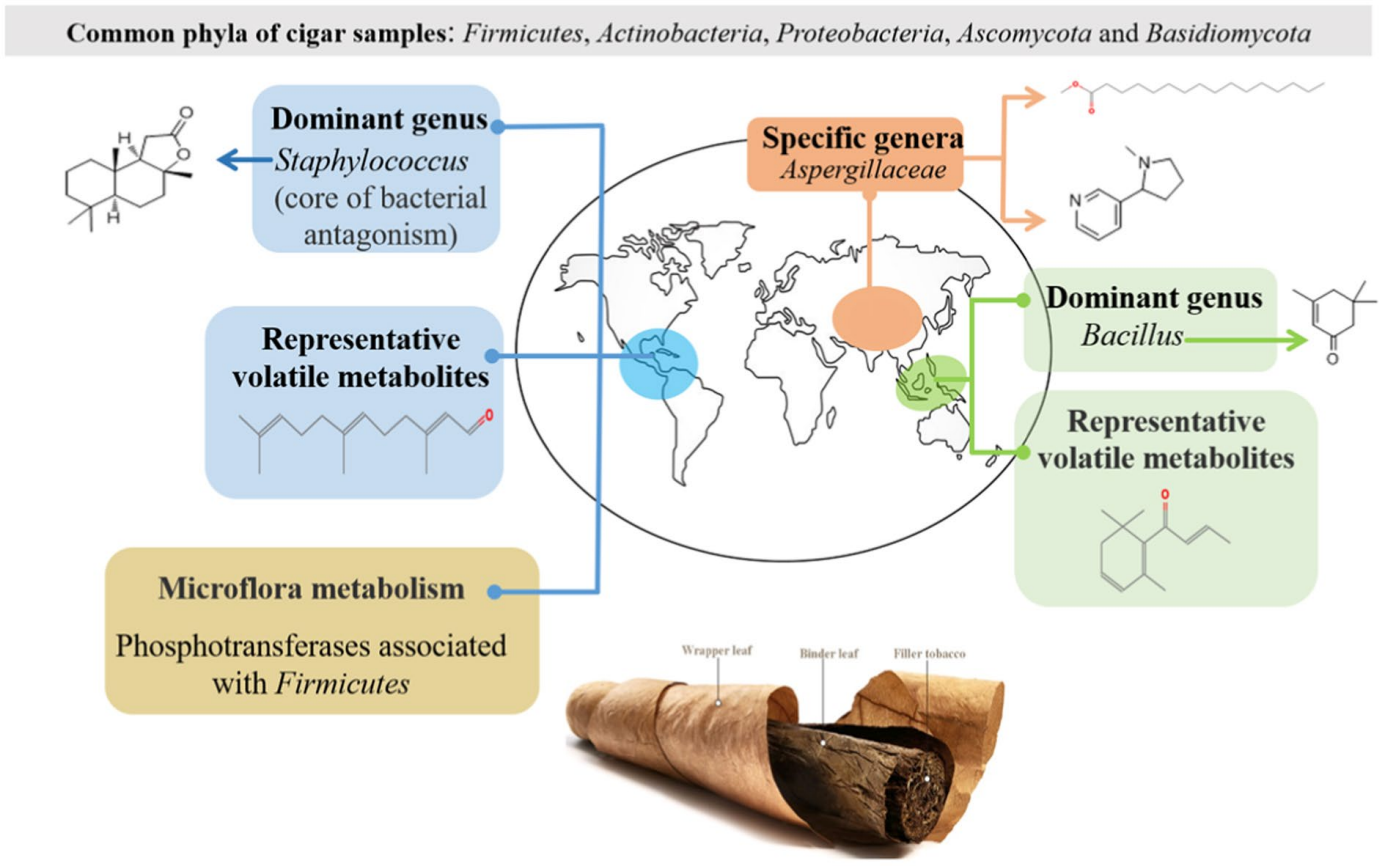

## Introduction

Tobacco is one of the largest non-food crops in the world. Cigar as one of its various commercialized products, widely popular in North America and around the world (Ferreira et al., 2019). According to the difference of tobacco leaf cultivation, they are divided into filler tobacco, binder leaf, and wrapper leaf (Frankenburg and Gottscho, 2002), filler tobacco is grown in the sun while tobacco used for the outside wrappers of cigars is usually grown under cheesecloth coverings to ensure uniform leaf texture and color (Wikle, 2015).

Cigar fermentation is a process in which multiple microorganisms are involved throughout (Wang et al., 2021). As a naturally alkaline fermentation environment due to the presence of tobacco alkaloids (Jensen and Parmele, 2002), cigar fermentation is particularly worthy investigating since the alkaline environment may significantly impact the microbial community structure (Smyth et al., 2019). Owing to the development of sequencing technology, a lot of work has been done recently focusing on the microbial community in this fermentation process. The high-throughput sequencing results suggested that bacterial community composition is brand-related (Rooney et al., 2005; Chopyk et al., 2017; Smyth et al., 2017; Malayil et al., 2020), origin-related (Su et al., 2011; Yang et al., 2020; Xing et al., 2021; Ye et al., 2021) and fermentation process-related (Li et al., 2020; Zhang et al., 2020; Zhou et al., 2020, 2021; Liu et al., 2021). Recently, chattopadhyay et al. overviewed the bacterial and fungal microbial communities in commercial tobacco products, and 89 unique bacterial genera and 19 fungal genera were identified in different tobacco products. *Bacillus*, *Pseudomonas*, and *Staphylococcus* are the commonly revealed dominant bacterial, while the fungal include *Aspergillus*, *Penicillium* (Chattopadhyay et al., 2021). Some researchers have also performed functional prediction of tobacco bacteria (Wu et al., 2016; Wang et al., 2018). Otherwise, the metabolic activities of tobacco microorganisms affect the formation of aromatic active compounds, which define the tobacco quality. To analyze the volatile metabolites in tobacco, gas chromatography–mass spectrometry (GC–MS) have been applied in recent studies as the most commonly used approach (Cai et al., 2013; Zhang et al., 2013; Lin et al., 2014; Qin et al., 2021; Vu et al., 2021), the main metabolites were often consisted of tobacco alkaloids, aldehydes, aldehydes and ketones, alcohols, alkane and alcohols (Nedeltcheva-Antonova et al., 2016; Farag et al., 2018; Tang et al., 2019; Zelinkova and Wenzl, 2021).

However, our understanding of cigar fermentation is still far from thorough. Although important information was provided by the recent multi-omics studies on tobacco product (Tsaballa et al., 2020; Tyx et al., 2020; Yan et al., 2020), the combinative analysis of metabolomic and bacterial-fungal microbial diversity are still extremely scarce. Moreover, current researches are mainly limited to focus on the same region, while the characteristics of cigars fermented in different regions may vary in a large extent.

Therefore, in this study, we collected cigar samples from four different regions, including the Caribbean, South America, Southeast Asia, and East Asia, aiming to provide regional interpretation of cigar fermentation. The community structure characteristics of these samples were compared and analyzed, and their metabolite composition was systematically analyzed, finally, the main microorganisms in cigars were correlated with the main metabolites of cigar fermentation. Results of the study are helpful to provide theoretical support of microbial community characteristic and metabolic pattern in alkaline fermentation environments.

## Materials and methods

### Source of cigar tobacco

All cigar samples were collected by Sichuan Provincial Branch of China National Tobacco Crop Tobacco Science Institute. Cigar tobacco samples were all superior to grade II after fermentation, 10 types of cigar leaves were collected from four different regions, including the Caribbean, South America, Southeast Asia, and East Asia (Table 1), technical triplicates were done per sample. Filler tobacco, binder leaf, and wrapper leaf was distinguished by the structural parts of cigar leaves (Frankenburg and Gottscho, 2002). Central position of leaves was uniformly selected from complete cigar tobacco leaves in each region. Samples for community and volatile metabolites analysis were stored at-80°C after the cigar tobacco leaves have been ground to a powder.

**Table 1:** The information of typical cigar samples from four regions.

Sample	Region	Position	Type
Carib_F	Caribbean	Central	Filler tobacco
Carib_B	Caribbean	Central	Binder leaf
Carib_W	Caribbean	Central	Wrapper leaf
SEA_F	Southeast Asia	Central	Filler tobacco
SEA_B	Southeast Asia	Central	Binder leaf
SEA_W	Southeast Asia	Central	Wrapper leaf
SA_F	South America	Central	Filler tobacco
EA_F	East Asia	Central	Filler tobacco
EA_B	East Asia	Central	Binder leaf
EA_W	East Asia	Central	Wrapper leaf

### Microbial community analysis

Three biological replicates for bacterial and fungal diversity analysis on cigar tobacco sample. Purified amplicons were pooled in equimolar and paired-end sequenced on an Illumina Mi Seq PE300 platform (Illumina, San Diego, United States) according to the standard protocols by Majorbio Bio-Pharm Technology Co. Ltd. (Shanghai, China). DNA purity detection method: NanoDrop2000, DNA concentration detection method: NanoDrop2000, DNA integrity detection method: agarose gel electrophoresis. For high-throughput sequencing, the experimental cigar tobacco was crushed in a wall breaker and screened through a 40-mesh sieve for reserve. Microbial community genomic DNA was extracted from 60 samples using the E.Z.N.A.® soil DNA Kit (Omega Bio-tek, Norcross, GA, U.S.) according to manufacturer’s instructions. The DNA extract was checked on 1% agarose gel, and DNA concentration and purity were determined with NanoDrop 2000 UV–vis spectrophotometer (Thermo Scientific, Wilmington, United States). The hypervariable region V3-V4 of the bacterial 16S rRNA gene were amplified with primer pairs 338F (5′-ACTCCTACGGGAGGCAGCAG-3′) and 806R (5′-GGACTACHVGGGTWTCTAAT-3′) by an ABI GeneAmp 9,700 PCR thermocycler (ABI, CA, United States). The hypervariable region ITS1F-ITS2R of the fungus gene were amplified with primer pairs ITS1F (5′-CTTGGTCA TTTAGAGGAAGTAA-3′) and ITS12R (5’-GCTGCGTTCTTCATCGATGC-3′). The PCR product was extracted from 2% agarose gel and purified using the AxyPrep DNA Gel Extraction Kit (Axygen Biosciences, Union City, CA, United States) according to manufacturer’s instructions and quantified using Quantus Fluorometer (Promega, United States).

The raw 16S rRNA and ITS gene sequencing reads were demultiplexed, quality-filtered by fastp version 0.20.0 (Chen et al., 2018) and merged by FLASH version 1.2.7 (Magoč and Salzberg, 2011). Operational taxonomic units (OTUs) with 97% similarity cutoff (Edgar, 2013) were clustered using UPARSE version 7.1 (Stackebrandt and Goebel, 1994), and chimeric sequences were identified and removed. The taxonomy of each OTU representative sequence was analyzed by RDP Classifier version 2.2 (Wang et al., 2007) against the 16S rRNA and ITS database using confidence threshold of 0.7. The raw reads were deposited into the NCBI Sequence Read Archive (SRA) database under the BioProject accession number PRJNA824254.

### Volatile metabolites analysis

Gas chromatography-mass spectrometer (GC–MS) was used to analyze volatile metabolites of tobacco leaves. Weighed 0.5 g of tobacco powder precisely, Chromatographic pure standards acetic acid 2-phenylethyl ester were added. Sample pre-treatment methods for extracting volatile metabolites were headspace solid-phase microextraction (HS-SPME), equilibrated at 70°C for 20 min and extracted for 30 min using a SPME fiber (50/30 μm DVB/CAR/PDMS, StableFlex 24 Ga, Sigma-Aldrich, Shanghai, China). GC–MS conditions were: DB-5 ms (30 m × 0.25 mm × 0.25 μm) chromatographic column, high purity helium carrier gas at a flow rate of 1.5 ml/min, 1 μl of injection volume, 240°C of injection temperature, injection method with split ratio of 5. EI source with ionization voltage 70 eV, 210°C of ion source temperature, 240°C of transmission line temperature, 3 min of solvent delay time, full scanning range: 33–350 AMU. Temperature programming method as shown below, selected 60°C as the initial temperature and kept for 2 min, then set 240°C at 4°C/min, kept for 5 min.

### Statistical analysis

Three replicate samples for statistical analyses. Analysis of microbial community differences between cigar samples from four regions using principal co-ordinates analysis, mapping based on the selected distance matrix, the distance algorithm was bray-curtis. The detected volatile metabolites were identified by comparing them with MS library of National Institute for Standards and Technology (NIST), using the semi-quantified peak area normalization method. Analysis of compounds with SI and RSI > 750 in MS Library. Differences in volatile metabolites between cigar samples were shown by heatmap, correlations between metabolites and microbial were calculated by R software (version 4.1.2) and then plotted (Hoyles et al., 2018). Interaction of microbial communities were demonstrated by the Gephi (version 0.9.2201709241107). In addition, the following software were used to analyze volatile metabolite data, including origin software (version 9.6.5.169 Origin Lab Corporation, Massachusetts, United States) and TB tools (Toolbox for Biologists version 0.6735). Gene functions were systematically analyzed through the KEGG database (Kyoto Encyclopedia of Genes and Genomes, http://www.genome.jp/kegg/), linking genomic, functional information, and enzymes. Triplicate in experiments were conducted on each sample to ensure good repeatability and the data was represented in the form of means ± relative standard deviation (RSD). One-way ANOVA with Duncan’s test was employed to investigate statistical differences. Correlation between metabolites and microbiota with *p* values of <0.05 (*n* = 3), were considered to be statistically significant.

## Results

### Analysis of microbial community

#### Diversity and abundance

To analyze the commonalities and differences of cigar tobacco samples from various regions, the Ace and Chao index were used to identify the community richness, whereas Shannon and Simpson index were used to evaluate the community diversity. The sequencing depths were adequate for all data, as suggested by the sparse curves, shown in Supplementary Figure S1, and the detailed index values were listed in Supplementary Table S1.

**Figure 1:**
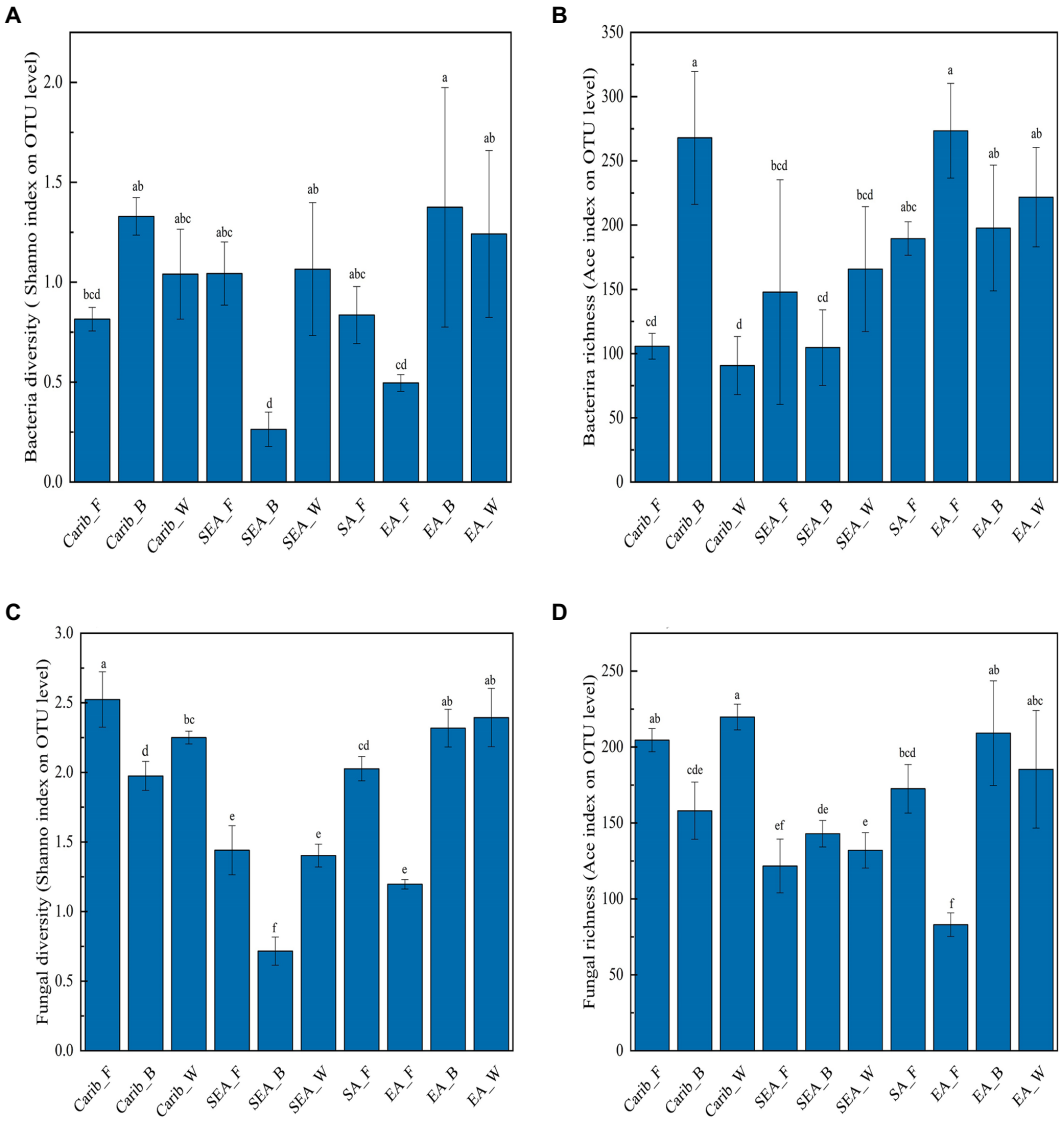
Microbial diversity and abundance analysis of cigar samples. **(A)** Bacterial diversity; **(B)** bacterial richness; **(C)** fungal diversity; and **(D)** fungal richness.

In the aspect of bacteria (Figures 1A,B), there was no general law describing neither the bacterial diversity nor richness. Carib_B and EA_B showed relatively high bacterial diversity while SEA_B exhibited the simplest bacterial community. Carib_B and EA_F were shown to have the highest bacterial richness among the samples. In the aspect of fungus (Figures 1C,D), no general law was revealed for neither the fungal diversity nor richness. The fungal richness of EA_F was demonstrated relatively low, and the results show that fungal diversity tightly depended on regions: the samples from SEA were shown with relatively low fungal diversity. It should be noted that both bacterial and fungal diversity were the lowest for the sample SEA_W, suggesting a relatively simple or pure microbial structure.

#### Community composition

In terms of the bacterial community composition for all the cigar samples, 476 OTUs were classified into 18 phyla, 41 classes, 97 orders, 158 families, 276 genera, and 385 species with 97% similarity.

**Figure 2:**
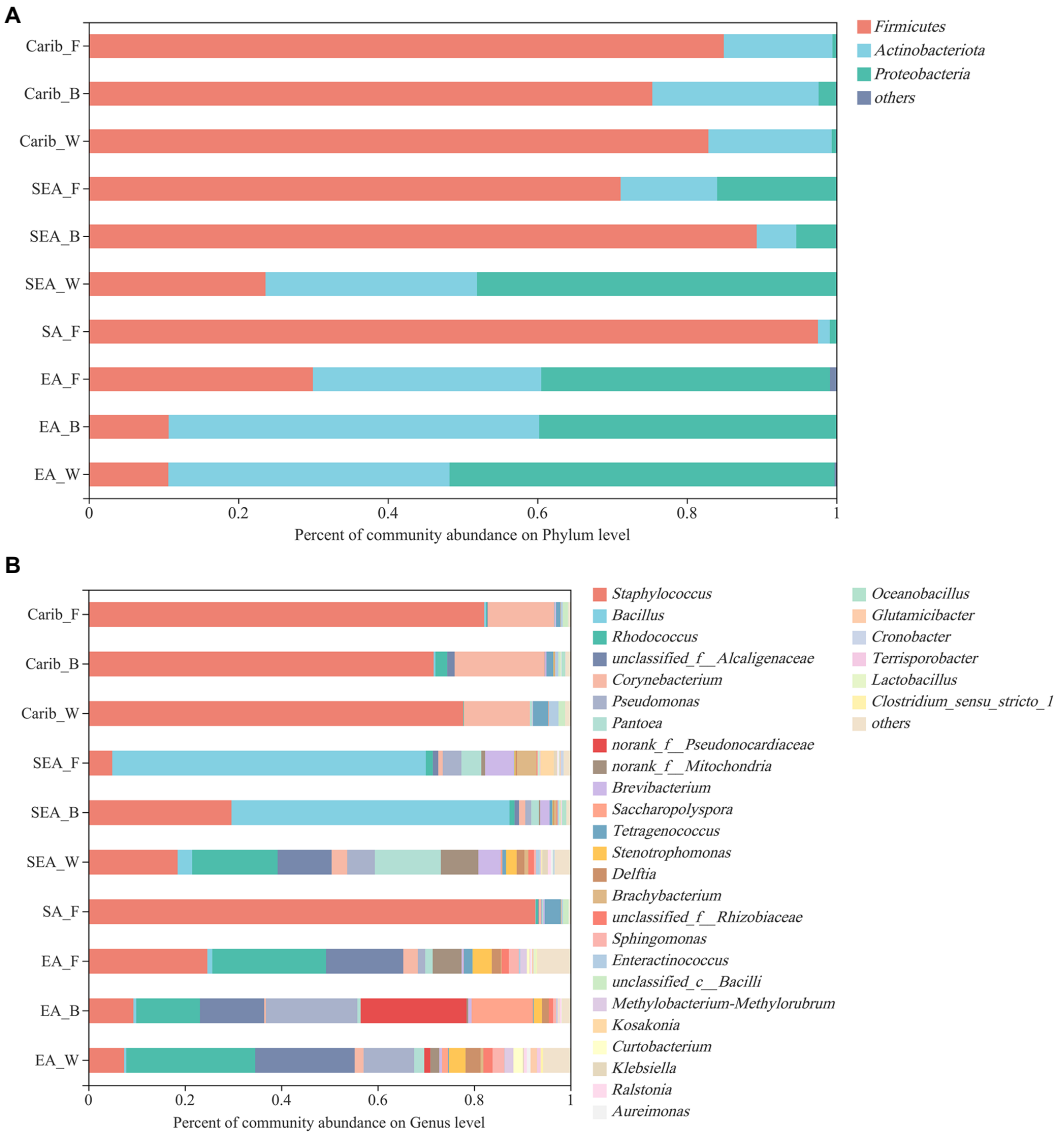
Relative abundances of major bacterial phylum **(A)** and genus **(B)** in cigar samples.

At the phyla classification level (Figure 2A), there were 3 bacterial phyla with a high average relative abundance, *Firmicutes* (10.65 to 97.5%), *Actinobacteria* (1.57 to 49.56%), and *Proteobacteria* (less than 51.49%) were the predominant phyla in these cigar samples. The results suggest that the dominant bacteria of cigar samples strongly depended on the continents of sampling. *Firmicutes* were the absolute dominant bacteria in the samples from the Americas (including South America and the Caribbean). In contrast, *Firmicutes* were no longer the dominant bacteria in samples from the Asian region (including Southeast and East Asia), especially for the samples from East Asia, where *Actinobacteria* and *Proteobacteria* accounted for a higher proportion of the bacterial community.

At the genus classification level (Figure 2B), it lists the top 30 bacterial genera with average relative abundance. The main bacterial genera were as follows: *Staphylococcus*, *Bacillus*, *Rhodococcus*, *Alcaligenaceae*, *Corynebacterium*, *Pseudomonas*, *Pantoea*, *Brevibacterium Saccharopolyspora*, *Tetragenococcus*, *Stenotrophomonas*, *Delftiza*, *Brachybacterium*, *Sphingomonas*, *Enteractinococcus*, *Methylobacterium-Methylorubrum*, *Kosakonia*, *Curtobacterium*, *Klebsiella, Oceanobacillus*, *Glutamicibacter* etc. *Staphylococcus* was the dominant genus in the Americas; *Bacillus* was the dominant genus in Southeast Asia; while in East Asia, there was no dominant genus. Otherwise, specific genera were demonstrated since they were identified only within certain samples: *Corynebacterium* within the samples of the Caribbean, *Bacillus* within the samples of Southeast Asia, and *Alcaligenaceae*, *Pseudonocardiaceae* within the samples of East Asia, which may contribute to their different volatile profiles, discussed in the metabolites section.

**Figure 3:**
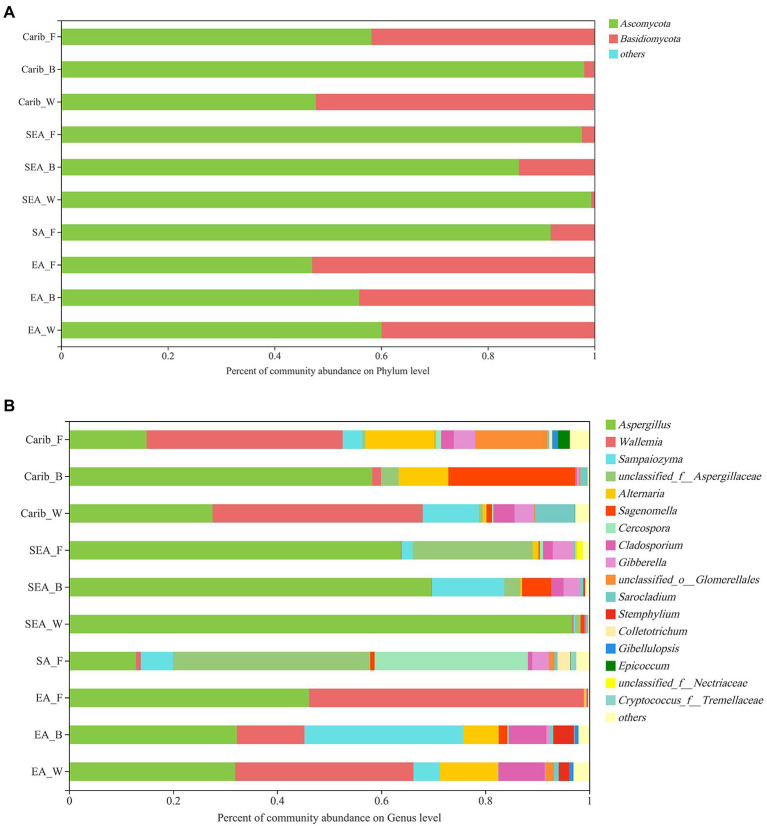
Relative abundances of major fungus phylum **(A)** and genus **(B)** in cigar samples.

As for the fungal community composition, shown in Figure 3, 870 OTUs were classified into 9 phyla, 29 classes, 67 orders, 157 families, 308 genera, and 490 species with 97% similarity.

At the phyla level of fungal composition (Figure 3A), there were 2 fungus phyla with a high average relative abundance, which were *Ascomycota* (47.05 to 99.35%), *Basidiomycota* (less than 52.93%). Both of them were the dominant phylum in different samples. To assess the overall distribution of the fungus community at the genus level (Figure 3B), 17 fungal genera with average relative abundance greater than 1% were selected, they were *Aspergillus*, *Wallemia*, *Sampaiozyma*, *Aspergillaccac*, *Alternaria*, *Sagenomella*, *Cercospora*, *Cladosporium*, *Gibberella*, *Glomerellales*, *Sarocladium*, *Stemphylium*, *Colletotrichum*, *Gibellulopsis*, *Epicoccum* etc. *Aspergillus* was the dominant fungal genus in all the cigar samples, especially in SEA samples. In addition to *Aspergillus*, *Wallemia* was also the dominant fungal genus in the Carib and EA samples. It is also notable that the SA samples had a higher percentage of *Cercospora* fungal genera than the other samples.

#### Clustering analysis

**Figure 4:**
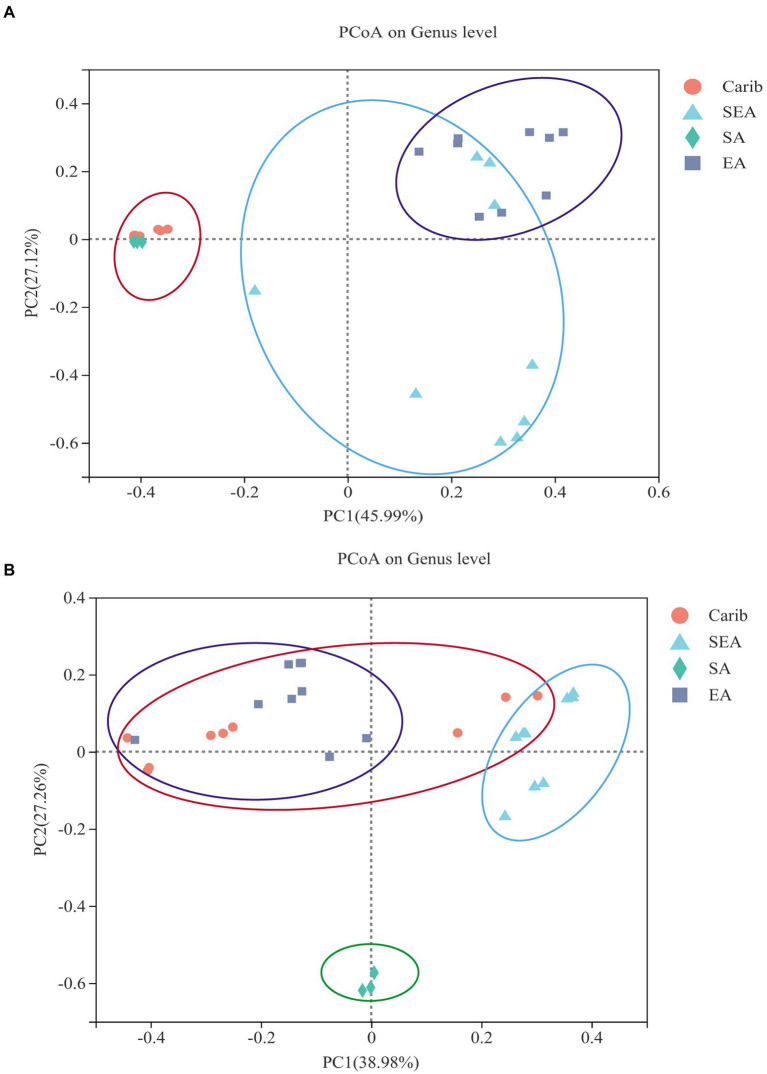
Principal co-ordinates analysis on genus level with cigar bacteria **(A)** and fungus **(B)**.

In order to illustrate the difference of microbial community among the samples from different typical regions more comprehensively, clustering analysis was carried out. Filler tobacco, binder leaf, and wrapper leaf of each region were divided into four separate groups. The PCoA plots were obtained *via* a comparative inter-group analysis of the diversity of tobacco microorganisms of different regions at the genus level, followed by a dimensionality reduction analysis. The results were representative since the bi-axial principal components of both Figures 4A,B covered more than 65% of the original data. As shown in Figure 4A, the microbial communities of cigar samples were strongly continental dependent: the samples from the Caribbean (Carib) and South America (SA) were demonstrated with high similarities, and samples from East Asia (EA) and Southeast Asia (SEA) were shown to share commonalities to a certain extent. As for the fungal communities, cigar samples from South America were independent from the other three regions, among which no tractable patterns could be identified between them (Figure 4B).

**Figure 5:**
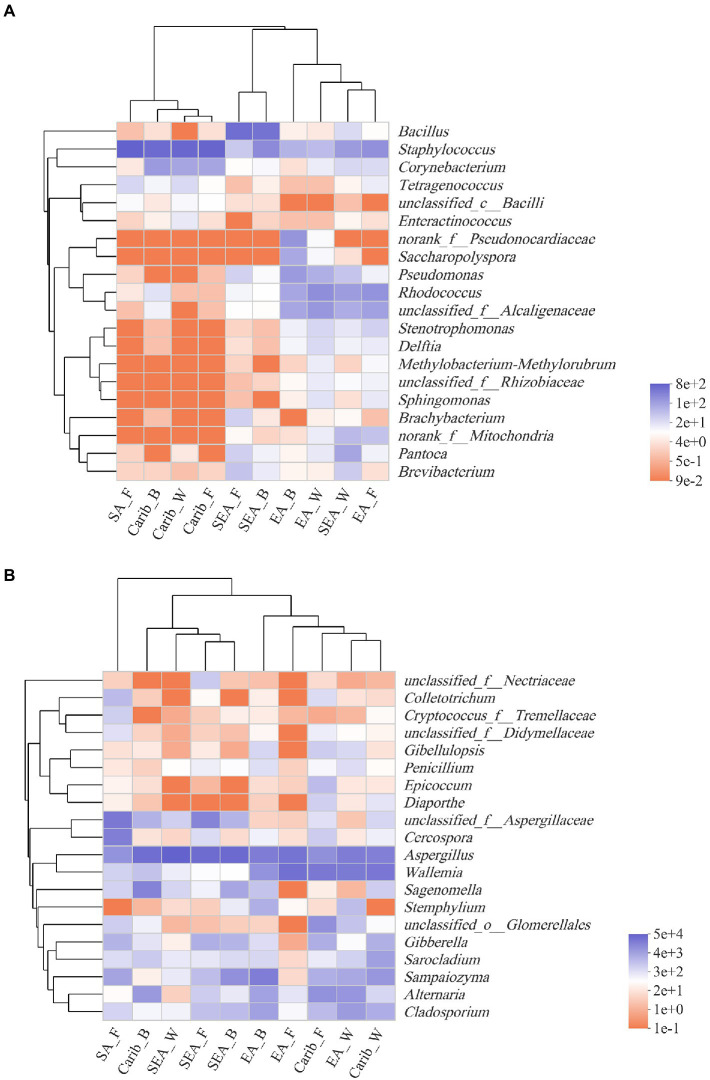
Heatmap of bacterial **(A)** and fungal **(B)** community at genus level based on cluster analysis.

The top 20 genera in terms of abundance were selected for further clustering, the results were presented on the heat-maps of bacterial and fungal community structure (Figure 5). The species with high and low abundance could be grouped together, the similarity and difference of community composition of different samples at different taxonomic levels could be reflected by color change and similarity degree. The redder the color, the lower the relative abundance of the genus in the sample, and the bluer the color, the higher the relative abundance of the genus in the sample.

It can be seen from the results of bacterial cluster analysis in Figure 5A, Carib_F, Carib_B, Carib_W, and SA_F samples clustered in a small branch, indicating that the bacterial community structure of these four samples from American continent was quite similar. It can be seen from the cluster analysis results of fungus in Figure 5B, there was no clustering between SA_F sample and other samples, indicating that the fungal community structure was significantly different from that of the other 9 samples. The different cultivation methods of tobacco from the SEA region clustered with Carib_B, suggesting a similar fungal composition in these regions.

### Interaction of microbial communities

**Figure 6:**
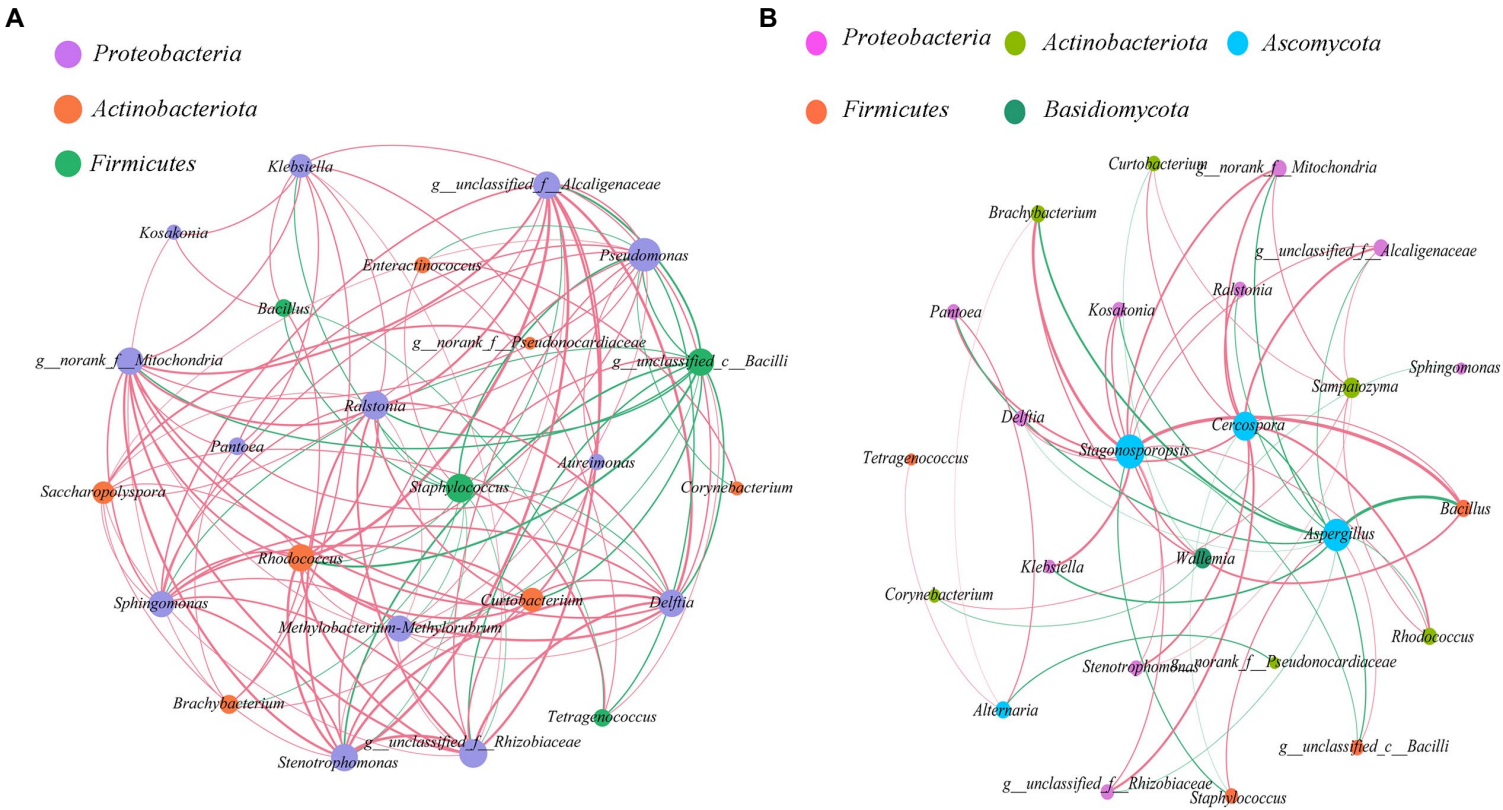
Co-occurrence and antagonism relationship among microbes within the cigar samples (|ρ| > 0.5, value of *p* <0.05). **(A)** bacterial-bacterial interaction; **(B)** bacterial-fungal interaction. Size of each node was proportional to the number of connections, the nodes were colored in groups, and the edge thickness was proportional to the absolute value of the spearman correlation coefficient.

The bacterial-bacterial and bacterial-fungal interaction of cigar leaves in different regions were studied by selecting 24 dominant bacteria and 5 fungal in this specific alkaline fermentation system (Figure 6). Red curve indicates positive correlation between two species while green curve indicates negative correlation. we studied the co-occurrence and co-exclusion patterns of microbial communities in tobacco leaves based on Spearman rank correlation (|ρ| > 0.5, value of *p* <0.05).

As for the bacterial-bacterial interaction (Figure 6A), it seems that the dominant *Staphylococcus* in the Caribbean and South America was the “core of bacterial antagonism” since it was shown negatively correlated with most of the selected *Proteobacteria* and *Actinobacteria* within the microflora. Meanwhile, the abundant *Rhodococcus* and *Alcaligenaceae* were the main “bacterial symbiotic centers” with strong positive correlation with most of the other bacteria.

As for the bacterial-fungal interaction (Figure 6B), the result confirmed that the dominant fungus *Aspergillus* was positively correlated with the dominant bacteria *Staphylococcus*. It also revealed that the dominant fungus *Aspergillus* was negatively correlated with the other abundant microorganisms. Interestingly, even not quite abundant within the microflora, the fungus *Cercospora* and *Stagonosporopsis* were demonstrated to positively correlate with several microorganisms, playing the role of “fungal symbiotic centers” within the microflora.

### Analysis of volatile metabolites

**Figure 7:**
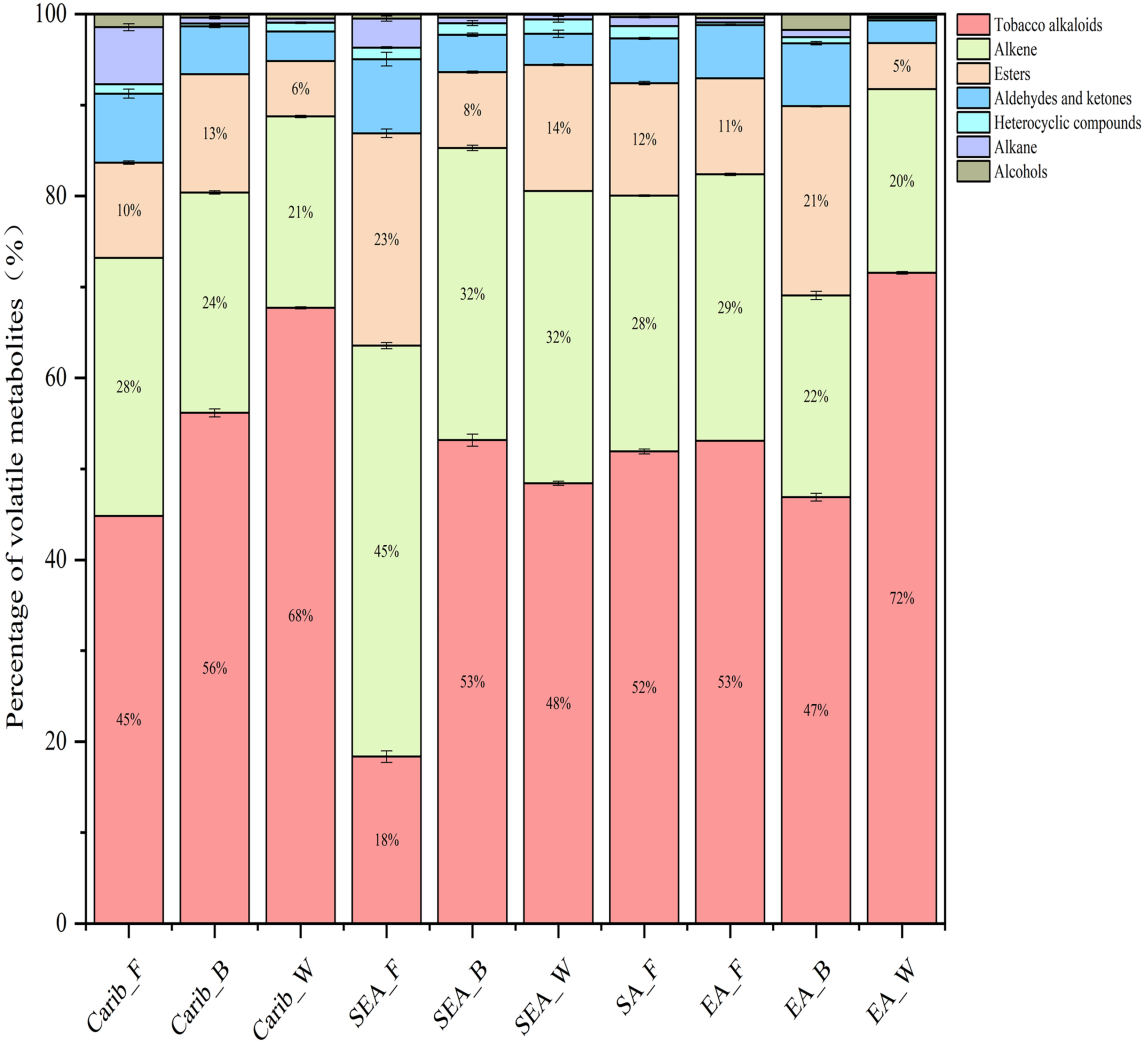
Composition of volatile metabolites of cigars from different regions.

A total of 69 to 169 compounds were detected in the analysis, detailed compound list for each sample can be found in supplementary (Supplementary Table S2). The criteria we selected are based on compounds with high detection frequency and academic research in the tobacco leaf fermentation system. In summary, the detected volatile metabolites can be divided into seven categories, including tobacco alkaloids, alkene, esters, aldehydes and ketones, heterocyclic compounds, alkane and alcohols. The first three categories were the major volatile metabolites in all samples, as shown in Figure 7. We also noted that the cigar cultivated in shade (wrapper leaf) tended to form more tobacco alkaloids, as found in both Caribbean (Carib_W) and East Asian (EA_W) samples. Among the many tobacco samples, SEA_F contained the least amount of tobacco alkaloids and the most esters.

**Figure 8:**
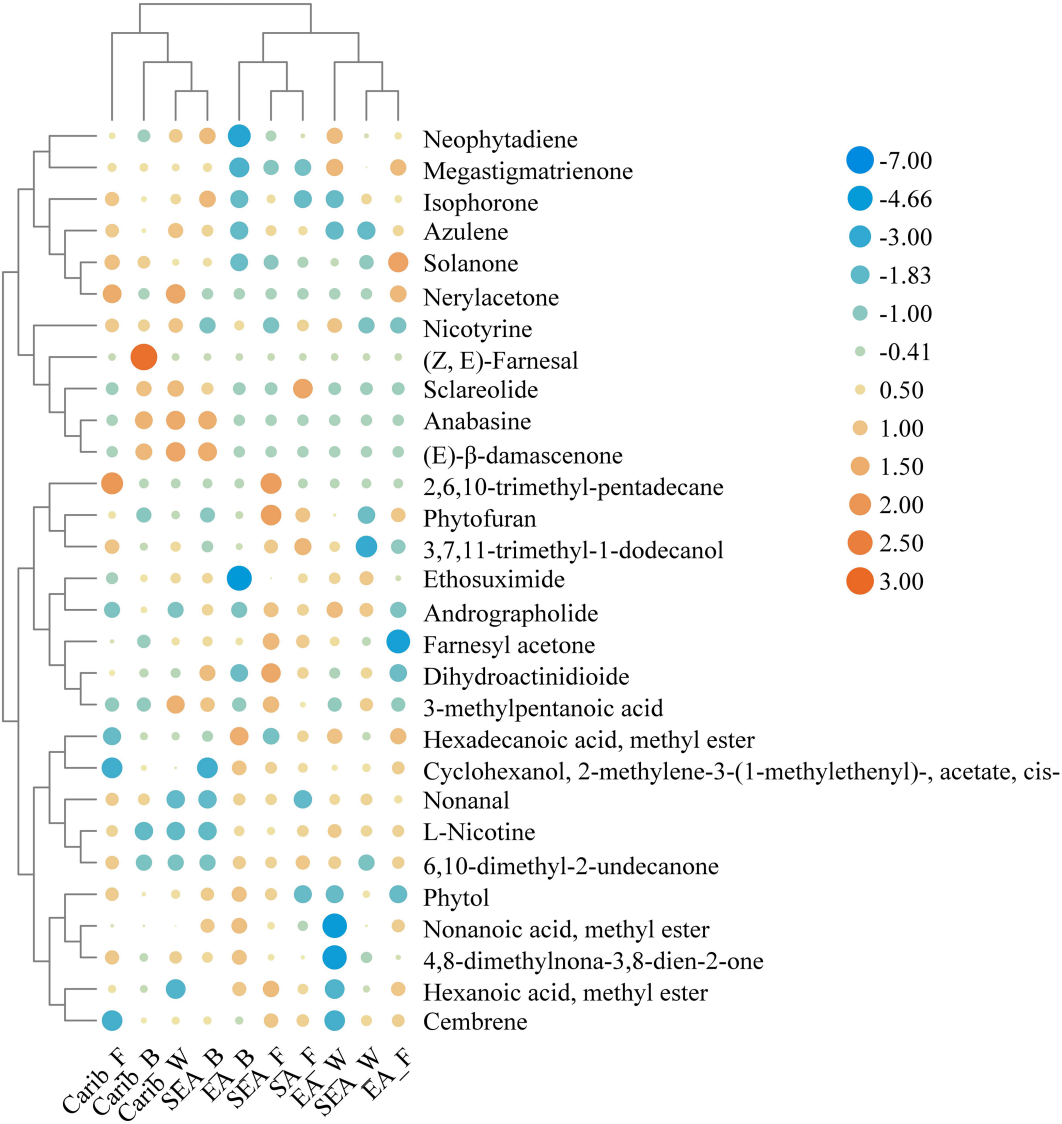
Heatmap of typical volatile metabolites in the cigar samples.

In order to further reveal the commonalities and differences of volatile profile among samples, 29 key volatile metabolites were selected as the target compounds for analysis, with the aim of identifying metabolites that differ the samples from different continents (Figure 8). The volatile metabolites among different continents also indicate certain patterns. Caribbean and SEA_B cigar samples have similar flavor profile, rich in certain substances such as (E)-β-damascenone, anabasine, nerylacetone, azulene, and isophorone. There were typical volatile metabolites in each region. (Z, E)-farnesal was only present in significant amounts in the binder leaf sample of Caribbean. Sclareolide was quite abundant in the samples of South America and the cigar samples of East Asia were rich in hexadecanoic acid, methyl ester.

### Correlation between metabolites and microbiota

So far, the dominant microorganisms of cigar samples from different regions were revealed, and the key metabolites were also identified. In this section, the correlation heat-map was built-up to better clarify the relationship between the outcome metabolites and the dominant microorganisms. The spearman correlation between the absolute abundance of metabolites and the relative abundance of microbes yielded several metabolite-microbe clusters (|ρ| > 0.5, value of *p* <0.05).

**Figure 9:**
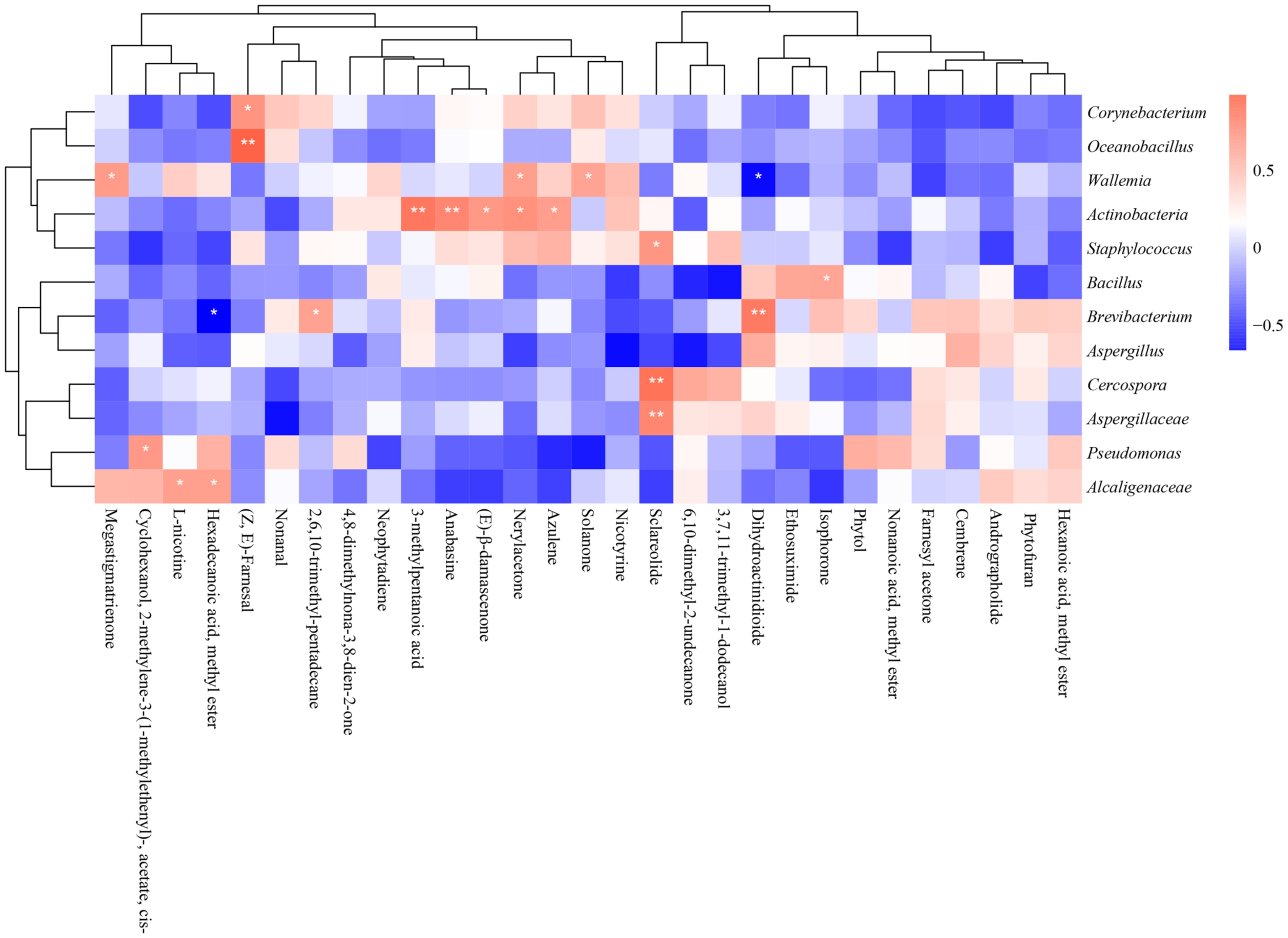
Heatmap of the correlation between dominant microorganisms and volatile metabolites in different continents (*p < 0.05, **p < 0.01). Blue represents negative correlation, red represents positive correlation.

Figure 9 illustrated the correlation between 29 typical volatile metabolites and 12 key microorganisms. It suggested that *Aspergillaceae*, *Cercospora*, and *Staphylococcus* were significantly correlated with Sclareolide. *Actinobacteria* were significantly associated with several metabolites: 3-methylpentanoic acid; anabasine; (E)-β-damascenone; Nerylacetone and azulene. *Bacillus* were positively associated with isophorone. *Alcaligenaceae* was significantly and positively correlated with L-nicotine and hexadecanoic acid, methyl ester. *Wallemia* were significantly correlated with solanone and megastigmatrienone. *Oceanobacillus* and *Corynebacterium* were positively associated with (Z, E)-farnesal.

### Prediction of microflora metabolism

**Figure 10:**
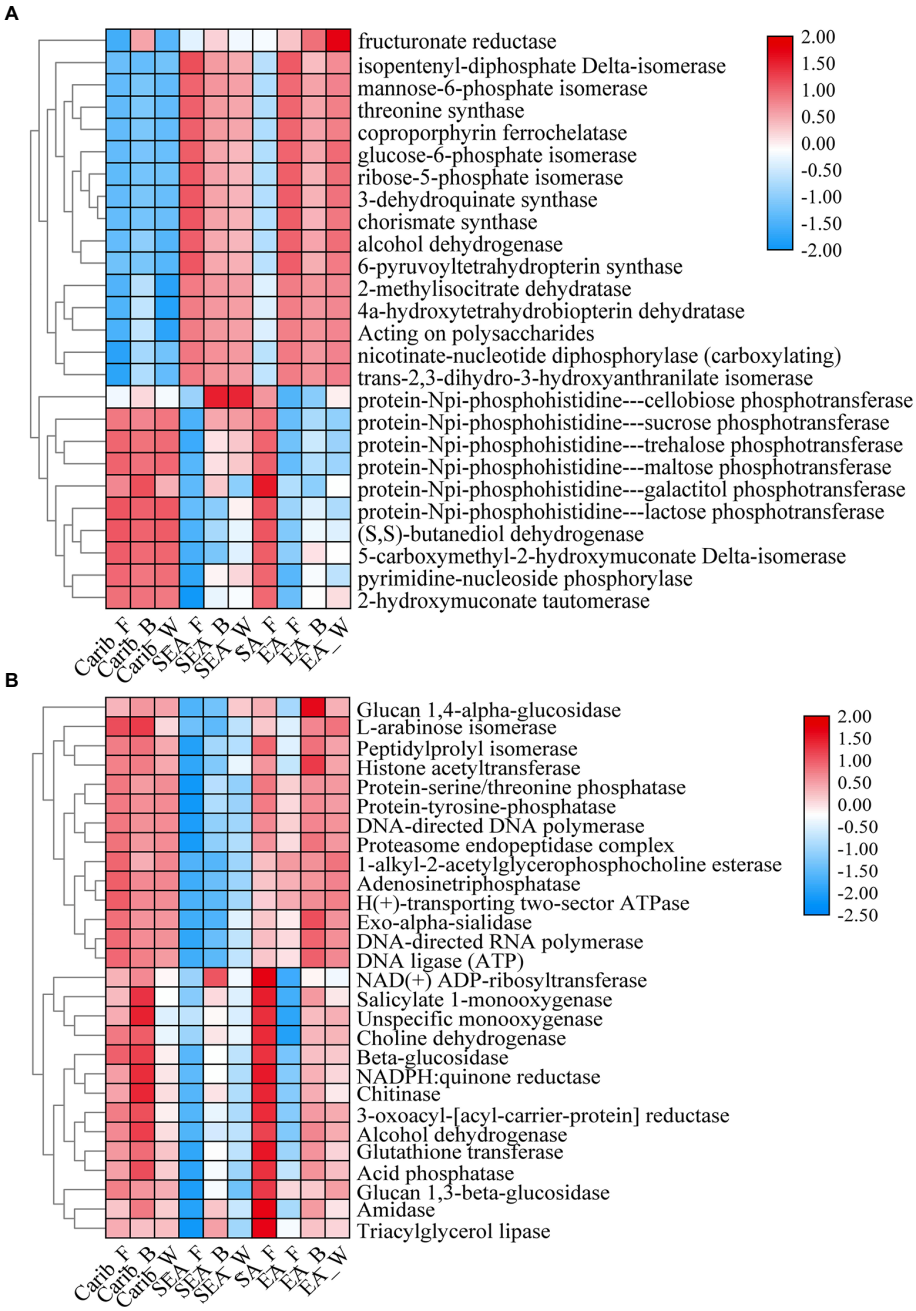
Functional heatmap of bacterial **(A)** and fungal **(B)** metabolic pathways.

The differential metabolic pathways and related enzymes were screened from Kyoto Encyclopedia of Genes and Genomes (KEGG) and represented as heat-maps, shown in Figure 10. The expression levels of bacterial-related enzymes were illustrated in Figure 10A. It again revealed a significant gap between the samples from the Americas and Asia: for example, the expression level of phosphotransferase was much more elevated for the samples from Americas (Carib, SA) than those from Asia (SEA, EA). Such difference in metabolism was related to their different microbial compositions.

Otherwise, as shown in Figure 10B, the expression level of fungal-related enzymes showed that there was no significant difference among the samples except for SEA. Obviously, the fungal-related enzymes expression was no longer continental dependent. It should be noted that the samples of SEA were already demonstrated as an exception in terms of fungal composition.

## Discussion

### Excavating microbial community characteristics of cigars from different regions

The role of microbe in influencing tobacco quality should not be underestimated. The bacteria communities of different cigar parts of tobacco grown in different regions vary greatly, While the fungal communities showed smaller differences. *Firmicutes*, *Actinobacteria*, *Proteobacteria* and *Ascomycota* were the dominant phyla (Huang et al., 2021). In addition, we found that based on the downscaling analysis and cluster analysis of bacterial and fungal communities, it all reflected that the bacterial community structure showed typical geographical characteristics with continental boundaries, while the fungal communities had no obvious geographical pattern, consistent with previous work (Xing et al., 2021). Microorganisms detected in cigar tobacco are involved in many fermentation-related stages, *Staphylococcus*, *Corynebacterium*, and *Oceanobacillus* were proved to have a positive effect on fermented products (Jia et al., 2021; Dias et al., 2022; Gao et al., 2022; Liu et al., 2022; Mhatre et al., 2022; Zhou et al., 2022; Li et al., 2023), *Corynebacterium* and *Oceanobacillus* also had alkaline resistance, which may be the reason why it could be the representative genus in fermentation (Hirota et al., 2013; Pang et al., 2020), which was related to the changes of aroma substances in cigar leaves after fermentation.

The inter-microbial association networks on cigars were also vital to the quality of tobacco (Zheng et al., 2022b). Studies have shown that 6,10-dimethyl-2-undecanone and farnesyl acetone in cigars were significantly positively correlated with floral and soybean aromas, respectively. Certain studies have also reported their effects on volatile flavor, such as correlation between *Acinetobacter* and production of major carbonyl compounds in cigar tobacco leaves (Zheng et al., 2022b).

### Integrating volatile metabolites and microbial of cigars from different regions

Volatile metabolites define the characteristics of cigar (Vu et al., 2021; Zheng et al., 2022a). These metabolites include endogenous and exogenous metabolites of cigar. Identification of the main metabolites in different cigar samples is particularly important to clarify the activities of microorganisms in different habitats. From the results of metabolite analysis, we noted the differences in volatile metabolites of cigars from different regions, cigar cultivated in shade (wrapper leaf) tended to form more tobacco alkaloids, as found in both Caribbean (Carib_W) and East Asian (EA_W) samples. It is worth noting that (Z, E)-farnesal was only present in significant amounts in the binder leaf sample of Caribbean. Otherwise, sclareolide was quite abundant in the samples of South America and the cigar samples of East Asia were rich in hexadecanoic acid, methyl ester. Meanwhile, the microbial could produce volatile metabolites by acting on tobacco leaves during the fermentation (Yan et al., 2020; Guo et al., 2021; Shimasaki et al., 2021). In this work we noticed that the *Actinobacteria* was positively correlated with aldehydes and ketones (Zheng et al., 2022b), which will help subsequent studies in improving tobacco quality.

For in-depth analysis of the difference among the cigar samples from different continents, microflora metabolism was predicted in this work. The expression level of phosphotransferase was much more elevated for the samples from Americas (Carib, SA) than those from Asia (SEA, EA). Such difference in metabolism was related to their different microbial compositions. Galactose was catalyzed by protein-Npi-phosphohistidine---galactitol phosphotransferase to form glycerone phosphate and D-glyceraldehyde 3-phosphate (Nolle et al., 2017; Schoenenberger et al., 2020; Huang et al., 2022; Zhao et al., 2022), associated with the *Firmicutes* (Saier et al., 2005; Yao et al., 2022, 2), which coincided with the high abundance of *Firmicutes* (*Staphylococcus*) on the characteristic of cigar bacteria in the Americas. Other sugar phosphotransferases mediated carbon and nitrogen metabolism (Wang et al., 2019), which associated with *Bacillus* and affected nitrogenous sugar content (Yoo et al., 2016), were also fit with the bacteria composition of the samples from the Americas. In addition, the expression level of (S, S)-butanediol dehydrogenase was also high in the samples from the Americas, which was also related to the high abundance of *Corynebacterium* (Jojima et al., 2015). This enzyme can catalyze the dehydrogenation of alcohols to aldehydes and ketones, and (Z, E)-farnesal were the representative compounds. The bacterial functional enzyme structures in Southeast Asia and Asia were relatively similar, mainly reflected in nicotinate-nucleotide diphosphorylase (carboxylating). Studies have shown that the nicotinate-nucleotide diphosphorylase (carboxylating) was isolated from *Alcaligenaceae* (Saunders and Bush, 1979; Schomburg and Stephan, 1996), which was associated with the abundance of bacteria in this region, pyridine compounds 5H-1-pyrindine, pyridine, 3-phenyl-and 2,3′-dipyridyl can be catalytically generated. As shown in the previous sections, the fungal diversity of these samples was relatively low (Figure 1C), and the absolute dominant fungus *Aspergillus* was revealed (Figure 3B). The results then suggest that the fungal autocracy may harm its metabolism in certain extents.

## Conclusion

Overall, in this work, Cigar samples from different regions (the Caribbeans, South America, East Asia, and Southeast Asia) were investigated, aiming to elucidate the key characteristics of this specific alkaline fermentation system. The results show that *Firmicutes*, *Actinobacteria*, *Proteobacteria, Ascomycota*, and *Basidiomycota* were the predominant phyla in the cigar samples. The microbial compositions were strongly depended on regions: *Staphylococcus* was the dominant genus in the Americas; *Bacillus* was the dominant genus in Southeast Asia; while in East Asia, there was no dominant genus. The bacterial community structure showed typical geographical characteristics with continental boundaries, while the fungal communities had no obvious geographical pattern. Such differences in community structure then affected the microflora metabolism: the expression level of phosphotransferase was quite different resulting from different abundancy of *Staphylococcus*. *Aspergillaceae*, *Cercospora*, and *Staphylococcus* were significantly correlated with sclareolide; *Alcaligenaceae* was significantly and positively correlated with L-nicotine and hexadecanoic acid, methyl ester. The results may provide valuable suggestions for cigar fermentation as well as other alkaline fermentation.

## Data availability statement

The datasets generated for this study can be found in the NCBI Sequence Read Archive (SRA, https://www.ncbi.nlm.nih.gov/sra/PRJNA824254) database under the BioProject accession number PRJNA824254.

## Author contributions

TL: investigation, formal analysis, and writing – original draft. SG: resources and project administration. CW: investigation and methodology. RuZ and QZ: resources and investigation. HS: project administration and conceptualization. RoZ: methodology. YQ: conceptualization and supervision. YJ: conceptualization, writing – review and editing, and supervision. All authors contributed to the article and approved the submitted version.

## Funding

This work was financially supported by the Key Research Project of Sichuan Provincial Branch of China National Tobacco Crop Tobacco Science Institute (SCYC202124).

## Conflict of interest

RuZ and QZ are employed by Deyang Tobacco Company.

The remaining authors declare that the research was conducted in the absence of any commercial or financial relationships that could be construed as a potential conflict of interest.

## Publisher’s note

All claims expressed in this article are solely those of the authors and do not necessarily represent those of their affiliated organizations, or those of the publisher, the editors and the reviewers. Any product that may be evaluated in this article, or claim that may be made by its manufacturer, is not guaranteed or endorsed by the publisher.
