# Supplementary material for: Phyllosphere microbial community of cigar tobacco and its corresponding metabolites
Source: Front Microbiol. 2022 Nov 11;13:1025881. doi: 10.3389/fmicb.2022.1025881 (PMC9691965; doi:10.3389/fmicb.2022.1025881)
Supplement: Supplementary file 1 [file Data_Sheet_1.docx]

Table S1 Index of bacterial and fungal diversities in cigar samples

| Sample | Richness index | | | | Diversity index | | | |
| --- | --- | --- | --- | --- | --- | --- | --- | --- |
|  | ACE | | Chao | | Simpson | | Shannon | |
|  | Bacteria | Fungus | Bacteria | Fungus | Bacteria | Fungus | Bacteria | Fungus |
| Carib_F | 105.74±10.01 | 204.57±7.61 | 97.42±14.49 | 204.70±17.73 | 0.66±0.02 | 0.16±0.05 | 0.81±0.06 | 2.52±0.20 |
| Carib_B | 267.90±51.73 | 158.04±18.85 | 237.99±50.07 | 146.36±13.86 | 0.48±0.04 | 0.21±0.02 | 1.33±0.09 | 1.97±0.10 |
| Carib_W | 90.67±22.56 | 219.78±8.51 | 87.02±36.16 | 213.84±8.00 | 0.59±0.11 | 0.20±0.05 | 1.04±0.22 | 2.25±0.05 |
| SEA_F | 147.90±87.41 | 121.67±17.70 | 119.14±41.27 | 114.26±14.54 | 0.51±0.10 | 0.44±0.07 | 1.04±0.16 | 1.44±0.18 |
| SEA_B | 104.69±29.46 | 142.92±8.69 | 102.46±4.70 | 107.17±7.65 | 0.93±0.03 | 0.72±0.04 | 0.26±0.09 | 0.72±0.10 |
| SEA_W | 165.77±48.59 | 132.00±11.67 | 147.27±25.75 | 125.06±17.24 | 0.54±0.20 | 0.42±0.05 | 1.07±0.33 | 1.40±0.08 |
| SA_F | 189.48±13.03 | 172.55±15.97 | 169.55±22.84 | 150.86±8.02 | 0.67±0.08 | 0.24±0.02 | 0.84±0.14 | 2.03±0.09 |
| EA_F | 273.41±36.86 | 83.02±7.84 | 220.01±23.86 | 82.41±7.59 | 0.86±0.02 | 0.39±0.02 | 0.50±0.04 | 1.20±0.03 |
| EA_B | 197.70±48.92 | 209.17±34.50 | 162.42±20.17 | 182.75±2.98 | 0.47±0.26 | 0.17±0.03 | 1.37±0.60 | 2.32±0.14 |
| EA_W | 221.74±38.75 | 185.31±38.74 | 210.04±55.39 | 165.62±16.11 | 0.59±0.08 | 0.17±0.06 | 1.24±0.42 | 2.39±0.21 |

Table S2 Analysis of volatile metabolites in cigar samples (Two consecutive tables)

| **Number** | **Name** | **Concentration (means±standard deviation，μg/Kg）** | | | | | |
| --- | --- | --- | --- | --- | --- | --- | --- |
|  |  | **Carib_F** | **Carib_B** | **Carib_W** | **SEA_F** | **SEA_B** | **SEA_W** |
|  | **Tobacco alkaloids (4)** |  |  |  |  |  |  |
| 1 | Anabasine | 0 | 90648.77±723.98 | 239721.68±230.70 | 0 | 141043.55±1549.82 | 0 |
| 2 | L-Nicotine | 92389.22±117.87 | 0 | 0 | 17487.24±604.91 | 0 | 74599.11±307.33 |
| 3 | Nicotyrine | 1098.56±1.40 | 564.50±4.66 | 1594.53±138.32 | 0 | 0 | 0 |
| 4 | Pyridine, 3-(3,4-dihydro-2H-pyrrol-5-yl)- | 93.74±0.12 | 0 | 558.51±66.00 | 0 | 514.20±244.68 | 108.47±53.69 |
|  | **Total** | **93581.52±119.39** | **91213.27±728.64** | **241874.72±435.02** | **17487.24±604.91** | **141557.75±1794.5** | **74707.58±361.02** |
|  | **Alkene (14)** |  |  |  |  |  |  |
| 5 | Neophytadiene | 58359.30±69.83 | 38891.43±321.17 | 74147.44±457.39 | 42085.46±533.65 | 84883.60±732.45 | 49097.88±115.83 |
| 6 | 1-Dodecene | 324.65±0.41 | 67.16±0.55 | 0 | 33.46±0.15 | 0 | 25.85±0.89 |
| 7 | 1-Pentadecene | 285.17±0.36 | 0 | 187.51±0.28 | 0 | 0 | 0 |
| 8 | 1,11-Dodecadiene | 78.22±0.10 | 0 | 0 | 0 | 0 | 0 |
| 9 | 1H-3a,7-Methanoazulene, octahydro-3,8,8-trimethyl-6-methylene-, [3R-(3. alpha. ,3a. beta.,7. beta.,8a. alpha.)]- | 64.55±0.08 | 0 | 0 | 0 | 0 | 0 |
| 10 | Limonene | 21.08±0.03 | 0 | 0 | 0 | 0 | 0 |
| 11 | Ethanone, 1-(2-methyl-1-cyclopenten-1-yl)- | 16.24±0.02 | 0 | 0 | 0 | 0 | 0 |
| 12 | 3,7,11-Trimethyl-2,4-dodecadiene | 0 | 144.47±1.19 | 139.54±11.29 | 0 | 0 | 0 |
| 13 | Cyclohexane, 1-ethenyl-1-methyl-2,4-bis(1-methylethenyl)-, [1S-(1. alpha. ,2. beta.,4. beta.)]- | 0 | 71.11±2.11 | 188.10±0.28 | 147.51±25.39 | 0 | 0 |
| 14 | Naphthalene, 1,2,3,5,8,8a-hexahydro- | 0 | 60.40±9.11 | 0 | 0 | 0 | 0 |
| 15 | Cembrene | 0 | 68.35±0.56 | 96.76±14.19 | 284.64±53.58 | 100.35±12.07 | 140.58±7.84 |
| 16 | 2-Octene, 2-methyl-6-methylene- | 0 | 16.56±0.14 | 0 | 22.75±1.82 | 0 | 39.91±0.42 |
| 17 | 1-Undecene, 4-methyl- | 0 | 11.64±0.10 | 0 | 0 | 0 | 0 |
| 18 | 1,3,5,7-Cyclooctatetraene | 0 | 11.18±0.09 | 0 | 0 | 15.44±0.17 | 13.94±0.48 |
|  | **Total** | **59149.21±70.83** | **39342.3±335.02** | **74759.35±483.43** | **42573.82±614.59** | **84999.39±744.69** | **49318.16±125.46** |
|  | **Esters (31)** |  |  |  |  |  |  |
| 19 | Acetic acid, 2-phenylethyl ester | 20000.00±0.00 | 20000.00±0.00 | 20000.00±0.00 | 20000.00±0.00 | 20000.00±0.00 | 20000.00±0.00 |
| 20 | Dihydroactinidioide | 447.99±221.39 | 480.36±37.82 | 517.53±106.94 | 902.25±302.90 | 1000.24±133.37 | 684.17±48.13 |
| 21 | 2,2,4-Trimethyl-1,3-pentanediol diisobutyrate | 260.66±12.79 | 0 | 137.84±5.17 | 48.32±14.19 | 171.02±57.37 | 48.06±16.88 |
| 22 | Isopropyl palmitate | 207.97±61.08 | 0 | 0 | 0 | 0 | 0 |
| 23 | Di-n-octyl phthalate | 150.58±19.65 | 0 | 0 | 0 | 0 | 0 |
| 24 | Methyl dehydroabietate | 124.89±16.34 | 0 | 0 | 0 | 0 | 0 |
| 25 | Decanoic acid, 2-ethylhexyl ester | 98.40±0.13 | 0 | 0 | 0 | 0 | 0 |
| 26 | 1,2-Benzenedicarboxylic acid, dinonyl ester | 98.10±0.13 | 0 | 0 | 0 | 0 | 0 |
| 27 | Hexanedioic acid, dioctyl ester | 76.80±0.10 | 0 | 0 | 0 | 0 | 0 |
| 28 | Octadecanoic acid, 2-oxo-, methyl ester | 66.75±0.09 | 0 | 0 | 0 | 0 | 0 |
| 29 | Hexadecanoic acid, methyl ester | 63.52±4.38 | 132.35±1.09 | 124.46±7.43 | 87.06±15.81 | 115.26±4.80 | 108.78±28.73 |
| 30 | Valtrate | 69.86±3.30 | 0 | 49.27±0.07 | 28.10±2.25 | 0 | 0 |
| 31 | Isopropyl myristate | 49.77±8.12 | 0 | 0 | 0 | 0 | 0 |
| 32 | Benzeneacetic acid, methyl ester | 36.84±10.06 | 70.27±0.58 | 158.27±54.01 | 64.39±16.29 | 0 | 66.30±35.37 |
| 33 | Hexanoic acid, methyl ester | 26.51±4.46 | 8.72±0.07 | 0 | 120.88±9.68 | 15.39±0.22 | 8.98±0.28 |
| 34 | Nonanoic acid, methyl ester | 22.13±1.67 | 22.42±3.08 | 28.46±0.04 | 33.95±10.45 | 71.74±24.49 | 33.69±5.49 |
| 35 | Octanoic acid, methyl ester | 20.78±0.19 | 7.97±0.07 | 0 | 38.79±12.94 | 22.91±1.34 | 12.27±0.42 |
| 36 | Butanoic acid, phenylmethyl ester | 19.26±1.90 | 0 | 0 | 0 | 0 | 0 |
| 37 | Methyl valerate | 17.88±0.02 | 0 | 32.38±0.05 | 99.81±6.68 | 0 | 0 |
| 38 | 3-Oxabicyclo [4.1.0] heptan-2-one, 4,4,7,7-tetramethyl- | 0 | 93.33±0.77 | 0 | 0 | 0 | 0 |
| 39 | Sclareolide | 0 | 53.41±0.44 | 78.15±8.80 | 0 | 22.94±1.08 | 0 |
| 40 | Cyclohexanol, 2-methylene-3-(1-methylethenyl)-, acetate, cis- | 0 | 44.68±0.37 | 25.72±0.04 | 100.48±8.05 | 0 | 0 |
| 41 | Methyl tetradecanoate | 0 | 43.82±0.36 | 38.89±0.06 | 172.38±18.80 | 49.66±10.75 | 52.24±9.68 |
| 42 | Tetradecanoic acid, 12-methyl-, methyl ester, (S)- | 0 | 36.40±0.30 | 67.29±6.95 | 0 | 75.29±10.51 | 0 |
| 43 | cis-5,8,11,14,17-Eicosapentaenoic acid | 0 | 33.80±0.28 | 0 | 0 | 0 | 0 |
| 44 | 2H-Pyran-2-one, 5,6-dihydro-6-pentyl- | 0 | 20.64±0.17 | 0 | 0 | 0 | 0 |
| 45 | Andrographolide | 0 | 17.54±0.14 | 0 | 75.13±14.85 | 40.90±3.41 | 65.14±2.24 |
| 46 | Dodecanoic acid, isooctyl ester | 0 | 14.25±0.12 | 0 | 0 | 0 | 0 |
| 47 | Acetic acid, phenylmethyl ester | 0 | 12.25±0.10 | 0 | 0 | 0 | 0 |
| 48 | 9-Octadecenoic acid (Z)-, methyl ester | 0 | 11.14±0.09 | 0 | 0 | 0 | 0 |
| 49 | 2,6,10-trimethylundecanoic Acid, 2,2,2- trifluoroethyl ester | 0 | 10.65±0.09 | 0 | 0 | 0 | 0 |
|  | **Total** | **21858.69±365.8** | **21114±45.94** | **21258.26±189.56** | **21771.54±432.89** | **21585.35±247.34** | **21079.63±147.22** |
|  | **Aldehydes and ketones (48)** |  |  |  |  |  |  |
| 50 | Solanone | 3421.70±35.52 | 3281.62±360.50 | 2515.58±75.08 | 1417.62±18.53 | 2501.15±149.87 | 1438.53±8.08 |
| 51 | 1,6-Dioxacyclododecane-7,12-dione | 2645.14±1054.04 | 0 | 0 | 0 | 0 | 165.63±5.71 |
| 52 | Nerylacetone | 2568.57±946.32 | 0 | 2595.10±37.99 | 0 | 0 | 0 |
| 53 | 6,10-dimethyl-2-undecanone | 1722.62±2.20 | 0 | 0 | 824.51±1.25 | 0 | 0 |
| 54 | Megastigmatrienone | 1904.34±23.48 | 1501.18±487.54 | 1877.59±179.88 | 372.35±101.38 | 2131.04±319.33 | 1008.35±273.39 |
| 55 | 2-Decanone, 5,9-dimethyl- | 725.32±0.93 | 0 | 0 | 0 | 0 | 0 |
| 56 | Nonanal | 720.76±0.92 | 487.62±4.03 | 0 | 367.94±70.49 | 0 | 369.50±136.48 |
| 57 | 3-Decen-5-one | 392.85±0.50 | 0 | 0 | 0 | 0 | 0 |
| 58 | 2-Butanone, 4-(2,2-dimethyl-6-methylenecyclohexyl)- | 503.65±4.52 | 156.61±15.89 | 425.76±0.64 | 220.60±111.17 | 0 | 116.74±17.47 |
| 59 | 4-Octanone | 331.65±0.42 | 0 | 0 | 0 | 0 | 0 |
| 60 | Farnesyl acetone | 207.52±5.25 | 131.59±1.09 | 291.68±16.17 | 425.49±85.65 | 338.24±43.72 | 150.57±29.84 |
| 61 | 3,5,9-Undecatrien-2-one, 6,10-dimethyl-, (E, E)- | 209.17±14.55 | 0 | 257.18±4.41 | 129.28±20.96 | 156.77±32.78 | 0 |
| 62 | Bicyclo[3.1.0]hexan-3-one, 4-methyl-1-(1-methylethyl)- | 144.06±6.04 | 0 | 0 | 0 | 0 | 0 |
| 63 | Undecanal, 2-methyl- | 121.81±3.73 | 0 | 0 | 0 | 0 | 0 |
| 64 | 3,7-Nonadien-2-one, 4,8-dimethyl- | 117.03±2.73 | 0 | 0 | 0 | 0 | 0 |
| 65 | 4,8-Dimethylnona-3,8-dien-2-one | 96.23±16.07 | 16.75±0.14 | 119.79±3.04 | 34.69±10.66 | 97.71±16.55 | 11.23±0.82 |
| 66 | Decanal | 145.72±0.19 | 46.53±1.60 | 80.87±7.59 | 201.38±27.07 | 109.81±19.60 | 80.59±2.69 |
| 67 | 2-Hexenal, (E)- | 69.84±0.09 | 0 | 0 | 0 | 0 | 0 |
| 68 | Glutaraldehyde | 57.34±0.07 | 0 | 0 | 0 | 0 | 0 |
| 69 | Hexanal | 56.33±0.07 | 21.19±0.17 | 57.89±50.25 | 0 | 166.42±1.82 | 0 |
| 70 | Isophorone | 44.55±0.06 | 12.99±0.11 | 21.98±2.15 | 17.51±1.40 | 83.00±13.16 | 19.73±5.96 |
| 71 | Benzaldehyde | 39.46±0.05 | 0 | 234.21±20.41 | 48.96±15.53 | 254.26±2.78 | 247.18±3.56 |
| 72 | 6-Ethyl-5,6-dihydro-2H-pyran-2-one | 29.31±0.04 | 10.16±0.08 | 458.68±64.86 | 0 | 0 | 0 |
| 73 | (E)-β-damascenone | 27.32±0.03 | 53.37±8.86 | 124.82±0.19 | 0 | 98.36±9.45 | 0 |
| 74 | 4-Hepten-3-one, 5-methyl- | 22.55±0.03 | 0 | 0 | 0 | 0 | 0 |
| 75 | 3,7-Nonadien-2-one, 4,8-dimethyl- | 117.03±2.73 | 0 | 0 | 0 | 0 | 0 |
| 76 | 2,6,6-Trimethyl-2-cyclohexene-1,4-dione | 16.67±0.02 | 30.63±0.25 | 54.10±0.08 | 0 | 102.45±1.12 | 0 |
| 77 | Acetophenone | 15.87±0.02 | 54.59±0.45 | 83.30±21.50 | 23.53±16.28 | 65.68±1.11 | 27.21±12.17 |
| 78 | Dehydromevalonic lactone | 13.44±0.02 | 0 | 50.97±0.82 | 0 | 0 | 29.53±1.02 |
| 79 | 2-Nonenal, (E)- | 12.97±0.02 | 0 | 0 | 0 | 216.74±2.37 | 0 |
| 80 | Butanal, 2-ethyl- | 11.30±0.01 | 9.57±0.08 | 0 | 0 | 0 | 25.02±1.96 |
| 81 | Dehydromevalonic lactone | 13.44±0.02 | 0 | 50.97±0.82 | 0 | 0 | 29.53±1.02 |
| 82 | trans-beta-Ionone | 0 | 418.50±3.46 | 702.96±46.28 | 0 | 0 | 104.00±3.58 |
| 83 | 3-Buten-2-one, 4-(2,2,6-trimethyl-7-oxabicyclo [4.1.0] hept-1-yl)- | 0 | 328.80±2.72 | 503.65±0.76 | 0 | 572.88±21.13 | 0 |
| 84 | Benzeneacetaldehyde | 0 | 225.20±61.91 | 26.22±0.46 | 14.50±1.16 | 0 | 70.74±14.83 |
| 85 | 1,3-Cyclohexanedione, 2-(2-propenyl)- | 0 | 177.72±19.85 | 124.99±0.19 | 37.76±15.22 | 0 | 114.18±15.94 |
| 86 | Solavetivone | 0 | 77.62±0.64 | 142.91±12.63 | 74.26±5.95 | 0 | 0 |
| 87 | 4,4-Dimethyl-cyclohex-2-en-1-ol | 0 | 68.25±0.56 | 0 | 0 | 0 | 0 |
| 88 | 7-Oxabicyclo [4.1.0] heptan-2-one, 4,4,6-trimethyl- | 0 | 67.89±0.56 | 0 | 0 | 0 | 0 |
| 89 | 2-Buten-1-one, 1-(2,6,6-trimethyl-1,3-cyclohexadien-1-yl)- | 0 | 48.41±0.24 | 116.78±3.81 | 0 | 92.44±6.30 | 0 |
| 90 | 6-Methyl-3,5-heptadiene-2-one | 0 | 85.21±0.70 | 81.88±13.54 | 74.95±11.90 | 91.88±1.00 | 50.16±1.73 |
| 91 | (Z, E)-Farnesal | 0 | 38.76±0.32 | 0 | 0 | 0 | 0 |
| 92 | 2-Piperidinon | 0 | 70.72±0.58 | 0 | 0 | 0 | 0 |
| 93 | Pentanal | 0 | 28.42±0.23 | 205.62±0.31 | 0 | 0 | 0 |
| 94 | Dodecanal | 0 | 21.50±0.18 | 0 | 44.33±3.55 | 107.29±1.17 | 0 |
| 95 | 2-Hexenal | 0 | 14.60±0.12 | 29.30±3.81 | 0 | 43.79±0.48 | 48.39±1.67 |
| 96 | 1,4-Cyclohexanedione, 2,2,6-trimethyl- | 0 | 13.58±0.11 | 24.53±1.53 | 10.95±0.88 | 25.08±0.27 | 9.85±0.34 |
| 97 | Hexadecanal | 0 | 13.49±0.11 | 102.11±0.15 | 32.85±2.63 | 103.80±1.13 | 67.87±2.34 |
|  | **Total** | **16525.56±2120.69** | **7513.07±973.08** | **11361.42±569.35** | **4373.46±521.66** | **7358.79±645.14** | **4184.53±540.6** |
|  | **Alkane (34)** |  |  |  |  |  |  |
| 98 | Decane, 1-(ethenyloxy)- | 908.04±152.08 | 118.53±0.98 | 0 | 0 | 0 | 0 |
| 99 | Hexadecane | 2783.90±82.86 | 363.34±19.40 | 644.64±24.21 | 843.72±43.38 | 648.43±52.62 | 366.08±41.12 |
| 100 | Octane, 2-chloro- | 688.08±34.07 | 0 | 0 | 0 | 0 | 0 |
| 101 | Tetradecane | 646.72±22.74 | 0 | 0 | 17.20±0.43 | 0 | 0 |
| 102 | Dodecane, 2,6,10-trimethyl- | 582.67±66.23 | 0 | 33.97±0.05 | 0 | 90.25±0.50 | 0 |
| 103 | Pentadecane, 2,6,10-trimethyl- | 546.31±45.10 | 0 | 0 | 385.06±8.63 | 0 | 0 |
| 104 | Hexadecane, 2,6,10,14-tetramethyl- | 486.26±38.51 | 0 | 0 | 0 | 0 | 0 |
| 105 | Dodecane, 4,6-dimethyl- | 460.48±55.42 | 0 | 0 | 0 | 0 | 0 |
| 106 | Octadecane | 419.12±2.01 | 0 | 84.32±8.60 | 124.77±9.99 | 0 | 0 |
| 107 | Oxirane, dodecyl- | 411.07±8.30 | 0 | 86.17±11.36 | 122.76±42.75 | 60.64±0.66 | 0 |
| 108 | Undecane, 2-methyl- | 396.09±11.85 | 0 | 0 | 0 | 0 | 0 |
| 109 | Dodecane | 631.73±12.36 | 30.18±0.66 | 165.59±40.66 | 166.49±37.66 | 157.04±49.75 | 91.76±26.49 |
| 110 | Eicosane, 2-methyl- | 344.08±34.40 | 15.42±0.13 | 0 | 49.65±3.98 | 0 | 0 |
| 111 | Decane, 3,7-dimethyl- | 368.32±1.40 | 0 | 0 | 0 | 77.94±0.85 | 0 |
| 112 | Nonadecane | 498.46±0.64 | 0 | 0 | 0 | 0 | 0 |
| 113 | Hexadecane, 2-methyl- | 664.32±46.63 | 0 | 0 | 0 | 0 | 0 |
| 114 | Heptadecane, 2,6-dimethyl- | 274.35±0.35 | 0 | 0 | 0 | 0 | 0 |
| 115 | Hexadecane, 3-methyl- | 228.96±0.29 | 0 | 0 | 0 | 0 | 0 |
| 116 | Decane, 5,6-dimethyl- | 213.73±0.27 | 0 | 0 | 0 | 0 | 0 |
| 117 | Hexadecane, 4-methyl- | 205.86±0.26 | 0 | 0 | 0 | 0 | 0 |
| 118 | 2-Methyltetracosane | 340.19±24.41 | 92.21±6.23 | 73.63±0.11 | 243.59±26.61 | 116.36±1.27 | 25.16±21.27 |
| 119 | Heptadecane | 180.84±0.23 | 0 | 0 | 0 | 42.08±0.46 | 0 |
| 120 | Tridecane, 3-ethyl- | 153.86±31.79 | 0 | 0 | 0 | 0 | 0 |
| 121 | Heptadecane, 3-methyl- | 163.46±0.21 | 0 | 0 | 35.86±2.87 | 0 | 0 |
| 122 | Octane, 4,5-dipropyl- | 126.82±0.16 | 0 | 0 | 0 | 0 | 0 |
| 123 | Tetradecane, 4-ethyl- | 105.01±0.13 | 0 | 77.33±0.12 | 24.38±1.95 | 0 | 0 |
| 124 | Undecane, 2,5-dimethyl- | 93.34±0.12 | 0 | 0 | 0 | 0 | 0 |
| 125 | Decane, 2,3,5,8-tetramethyl- | 72.69±0.09 | 0 | 148.69±0.22 | 0 | 0 | 0 |
| 126 | Decane | 70.54±0.93 | 65.44±0.54 | 48.68±12.06 | 44.09±0.68 | 0 | 77.98±14.93 |
| 127 | Tridecane | 48.38±0.06 | 0 | 0 | 0 | 0 | 0 |
| 128 | Dodecane, 1-cyclopentyl-4-(3-cyclopentylpropyl)- | 23.02±0.03 | 0 | 0 | 0 | 0 | 0 |
| 129 | Cyclohexane, 1-propenyl- | 22.91±0.03 | 0 | 0 | 0 | 0 | 0 |
| 130 | Tetradecane, 4-methyl- | 18.10±0.02 | 0 | 0 | 0 | 28.57±0.31 | 0 |
| 131 | Cyclohexane, methyl- | 12.02±0.02 | 0 | 0 | 0 | 0 | 0 |
|  | **Total** | **13189.73±674** | **685.12±27.94** | **1363.02±97.39** | **2057.57±178.93** | **1221.31±106.42** | **560.98±103.81** |
|  | **Alcohols (17)** |  |  |  |  |  |  |
| 132 | 1,2-Dihydrolinalool | 390.86±0.50 | 0 | 0 | 0 | 0 | 0 |
| 133 | 3,7,11,15-Tetramethyl-2-hexadecen-1-ol | 376.94±0.48 | 323.07±2.67 | 1110.80±2.55 | 0 | 349.66±6.50 | 0 |
| 134 | 1-Dodecanol, 3,7,11-trimethyl- | 286.47±0.37 | 156.95±1.30 | 243.21±13.64 | 305.62±53.71 | 143.69±11.42 | 77.40±4.89 |
| 135 | Cyclohexanol, 2-methyl-5-(1-methylethenyl)- | 283.97±0.36 | 0 | 0 | 0 | 0 | 0 |
| 136 | 1-Dodecanol | 281.13±0.36 | 0 | 0 | 0 | 0 | 0 |
| 137 | 1-Hexanol, 5-methyl-2-(1-methylethyl)- | 264.71±0.34 | 9.11±0.08 | 0 | 0 | 0 | 0 |
| 138 | 1H-Cycloprop[e]azulen-4-ol, decahydro-1,1,4,7-tetramethyl-, [1aR-(1a. alpha.,4. beta.,4a. beta. ,7. alpha.,7a. beta.,7b. alpha.)]- | 229.99±0.29 | 0 | 0 | 0 | 0 | 0 |
| 139 | trans-2-Dodecen-1-ol | 430.02±0.55 | 0 | 0 | 0 | 0 | 0 |
| 140 | 1-Hexadecanol | 126.93±0.16 | 0 | 0 | 0 | 0 | 0 |
| 141 | Phytol | 128.86±0.16 | 25.58±4.43 | 42.58±1.27 | 64.78±10.79 | 98.86±5.58 | 31.05±1.07 |
| 142 | 1-Tetradecanol | 89.98±0.11 | 0 | 0 | 0 | 0 | 0 |
| 143 | n-Tridecan-1-ol | 61.72±0.08 | 0 | 0 | 0 | 0 | 0 |
| 144 | 3-Tetradecyn-1-ol | 27.20±0.03 | 0 | 0 | 0 | 0 | 9.08±0.31 |
| 145 | Carveol | 18.70±0.02 | 0 | 0 | 0 | 0 | 0 |
| 146 | n-Nonadecanol-1 | 17.34±0.02 | 0 | 0 | 0 | 0 | 0 |
| 147 | 1-Octanol, 2,7-dimethyl- | 16.99±0.02 | 0 | 0 | 7.04±0.56 | 0 | 0 |
| 148 | 2,6,11-Tridecatrien-10-ol, 2,6,10-trimethyl- | 3031.75±3.85 | 21.46±0.18 | 0 | 0 | 0 | 0 |
|  | **Total** | **3261.8±4.14** | **536.17±8.66** | **1396.59±17.46** | **377.44±65.06** | **592.21±23.5** | **117.53±6.27** |
|  | **Heterocyclic compounds (21)** |  |  |  |  |  |  |
| 149 | 2,6-Di-tert-butyl-p-benzoquinone | 697.56±0.89 | 0 | 0 | 0 | 0 | 0 |
| 150 | n-Hexadecanoic acid | 0 | 0 | 0 | 0 | 0 | 0 |
| 151 | Anthracene, tetradecahydro- | 0 | 0 | 0 | 0 | 0 | 0 |
| 152 | Benzene, 1,3,5-tri-tert-butyl- | 0 | 0 | 0 | 0 | 0 | 0 |
| 153 | 3-(4,8,12-Trimethyltridecyl) furan | 0 | 85.95±0.71 | 129.21±3.90 | 474.71±6.28 | 83.18±4.45 | 70.88±0.00 |
| 154 | Nonanoic acid | 0 | 0 | 29.88±0.05 | 0 | 0 | 0 |
| 155 | 5H-1-Pyrindine | 0 | 0 | 0 | 0 | 0 | 0 |
| 156 | Butane, 1-propoxy- | 0 | 0 | 0 | 0 | 0 | 0 |
| 157 | Hexadecanedinitrile | 0 | 0 | 0 | 0 | 0 | 0 |
| 158 | Benzene, (ethoxymethyl)- | 0 | 0 | 0 | 0 | 0 | 0 |
| 159 | Butyl triacontyl ether | 0 | 0 | 0 | 0 | 0 | 0 |
| 160 | Naphthalene, 2,3,6-trimethyl- | 0 | 0 | 0 | 0 | 0 | 0 |
| 161 | 2-Hydroxyhexadecanoic acid | 0 | 0 | 0 | 0 | 0 | 0 |
| 162 | Azulene | 0 | 14.21±0.12 | 54.52±16.59 | 25.72±5.55 | 34.84±0.38 | 0 |
| 163 | 1,4,10,13-tetraoxa-7,16-dithiacyclooctadecane | 0 | 0 | 0 | 0 | 0 | 0 |
| 164 | Benzenamine, N-(1-methylethyl)- | 0 | 32.24±0.27 | 29.62±0.04 | 42.03±2.78 | 36.51±0.40 | 44.43±1.53 |
| 165 | Ethosuximide | 0 | 56.31±16.58 | 94.97±0.14 | 46.53±0.74 | 116.85±25.59 | 216.88±3.97 |
| 166 | 2-n-Butyl furan | 0 | 0 | 0 | 0 | 0 | 0 |
| 167 | Ethosuximide | 0 | 56.31±16.58 | 94.97±0.14 | 46.53±0.74 | 116.85±25.59 | 216.88±3.97 |
| 168 | Furan, 2-pentyl- | 0 | 10.21±0.08 | 0 | 0 | 0 | 0 |
| 169 | 3-methylpentanoic acid | 0 | 367.54±0.03 | 115.45±0.06 | 76.75±0.07 | 203.03±0.09 | 367.54±0.02 |
|  | **Total** | **697.56±0.89** | **622.77±34.37** | **548.62±20.92** | **712.27±16.16** | **591.26±56.5** | **916.61±9.49** |

| **Number** | **Name** | **Concentration (means±standard deviation，μg/Kg）** | | | |
| --- | --- | --- | --- | --- | --- |
|  |  | **SA_F** | **EA_F** | **EA_B** | **EA_W** |
|  | **Tobacco alkaloids (4)** |  |  |  |  |
| 1 | Anabasine | 0 | 0 | 0 | 0 |
| 2 | L-Nicotine | 91702.12±397.53 | 108150.32±10.26 | 48369.57±435.88 | 299402.00±738.56 |
| 3 | Nicotyrine | 0 | 0 | 296.14±5.43 | 1659.72±19.32 |
| 4 | Pyridine, 3-(3,4-dihydro-2H-pyrrol-5-yl)- | 226.15±71.15 | 0 | 0 | 192.68±1.01 |
|  | **Total** | **91928.27±468.68** | **91928.27±468.68** | **108150.32±10.26** | **301254.4±758.89** |
|  | **Alkene (14)** |  |  |  |  |
| 5 | Neophytadiene | 49227.48±6.43 | 58724.00±295.49 | 22464.47±446.77 | 83418.13±143.05 |
| 6 | 1-Dodecene | 25.81±0.68 | 0 | 0 | 0 |
| 7 | 1-Pentadecene | 0 | 0 | 0 | 0 |
| 8 | 1,11-Dodecadiene | 0 | 0 | 0 | 0 |
| 9 | 1H-3a,7-Methanoazulene, octahydro-3,8,8-trimethyl-6-methylene-, [3R-(3. alpha. ,3a. beta.,7. beta.,8a. alpha.)]- | 0 | 0 | 0 | 218.00±88.58 |
| 10 | Limonene | 0 | 0 | 0 | 0 |
| 11 | Ethanone, 1-(2-methyl-1-cyclopenten-1-yl)- | 0 | 0 | 0 | 0 |
| 12 | 3,7,11-Trimethyl-2,4-dodecadiene | 0 | 0 | 0 | 0 |
| 13 | Cyclohexane, 1-ethenyl-1-methyl-2,4-bis(1-methylethenyl)-, [1S-(1. alpha. ,2. beta.,4. beta.)]- | 181.59±4.81 | 0 | 0 | 0 |
| 14 | Naphthalene, 1,2,3,5,8,8a-hexahydro- | 0 | 0 | 0 | 0 |
| 15 | Cembrene | 193.93±12.85 | 208.78±0.09 | 19.97±0.37 | 0 |
| 16 | 2-Octene, 2-methyl-6-methylene- | 0 | 0 | 116.38±2.13 | 34.23±0.18 |
| 17 | 1-Undecene, 4-methyl- | 0 | 0 | 0 | 191.84±1.01 |
| 18 | 1,3,5,7-Cyclooctatetraene | 0 | 0 | 0 | 0 |
|  | **Total** | **49628.81±24.77** | **58932.78±295.58** | **22600.82±449.27** | **83862.2±232.82** |
|  | **Esters (31)** |  |  |  |  |
| 19 | Acetic acid, 2-phenylethyl ester | 20000.00±0.00 | 20000.00±0.00 | 20000.00±0.00 | 20000.00±0.00 |
| 20 | Dihydroactinidioide | 685.80±56.56 | 345.86±62.36 | 329.52±38.41 | 396.07±40.30 |
| 21 | 2,2,4-Trimethyl-1,3-pentanediol diisobutyrate | 34.78±0.92 | 24.67±0.00 | 48.02±12.15 | 58.89±0.31 |
| 22 | Isopropyl palmitate | 0 | 0 | 0 | 0 |
| 23 | Di-n-octyl phthalate | 0 | 0 | 0 | 0 |
| 24 | Methyl dehydroabietate | 0 | 0 | 0 | 0 |
| 25 | Decanoic acid, 2-ethylhexyl ester | 0 | 0 | 0 | 0 |
| 26 | 1,2-Benzenedicarboxylic acid, dinonyl ester | 0 | 0 | 0 | 0 |
| 27 | Hexanedioic acid, dioctyl ester | 17.94±0.48 | 0 | 0 | 0 |
| 28 | Octadecanoic acid, 2-oxo-, methyl ester | 0 | 0 | 0 | 0 |
| 29 | Hexadecanoic acid, methyl ester | 219.77±30.59 | 347.74±18.15 | 356.69±79.63 | 271.81±59.18 |
| 30 | Valtrate | 0 | 0 | 0 | 0 |
| 31 | Isopropyl myristate | 0 | 0 | 0 | 0 |
| 32 | Benzeneacetic acid, methyl ester | 141.74±19.32 | 53.77±21.97 | 0 | 0 |
| 33 | Hexanoic acid, methyl ester | 35.45±9.14 | 62.24±26.29 | 73.03±6.43 | 0 |
| 34 | Nonanoic acid, methyl ester | 12.01±1.16 | 66.22±18.23 | 140.77±24.84 | 0 |
| 35 | Octanoic acid, methyl ester | 0 | 36.76±12.63 | 50.70±11.04 | 0 |
| 36 | Butanoic acid, phenylmethyl ester | 0 | 0 | 0 | 0 |
| 37 | Methyl valerate | 0 | 12.07±0.00 | 11.33±0.21 | 0 |
| 38 | 3-Oxabicyclo [4.1.0] heptan-2-one, 4,4,7,7-tetramethyl- | 0 | 0 | 0 | 0 |
| 39 | Sclareolide | 157.99±42.27 | 0 | 0 | 0 |
| 40 | Cyclohexanol, 2-methylene-3-(1-methylethenyl)-, acetate, cis- | 104.70±30.41 | 0 | 189.31±3.47 | 0 |
| 41 | Methyl tetradecanoate | 177.01±50.69 | 170.23±27.47 | 176.77±40.96 | 109.98±4.84 |
| 42 | Tetradecanoic acid, 12-methyl-, methyl ester, (S)- | 0 | 24.74±0.00 | 9.74±0.00 | 0 |
| 43 | cis-5,8,11,14,17-Eicosapentaenoic acid | 0 | 0 | 0 | 0 |
| 44 | 2H-Pyran-2-one, 5,6-dihydro-6-pentyl- | 0 | 0 | 0 | 0 |
| 45 | Andrographolide | 44.38±3.61 | 0 | 0 | 121.52±0.64 |
| 46 | Dodecanoic acid, isooctyl ester | 0 | 0 | 0 | 0 |
| 47 | Acetic acid, phenylmethyl ester | 0 | 0 | 0 | 0 |
| 48 | 9-Octadecenoic acid (Z)-, methyl ester | 0 | 0 | 37.77±0.69 | 0 |
| 49 | 2,6,10-trimethylundecanoic Acid, 2,2,2- trifluoroethyl ester | 0 | 0 | 0 | 0 |
|  | **Total** | **21631.57±245.15** | **21144.3±187.1** | **21423.65±217.83** | **20958.27±105.27** |
|  | **Aldehydes and ketones (48)** |  |  |  |  |
| 50 | Solanone | 1595.93±115.33 | 4680.40±53.76 | 1199.12±5.95 | 1983.09±181.28 |
| 51 | 1,6-Dioxacyclododecane-7,12-dione | 0 | 165.37±0.00 | 0 | 0 |
| 52 | Nerylacetone | 0 | 669.16±1.52 | 0 | 0 |
| 53 | 6,10-dimethyl-2-undecanone | 2237.09±4.43 | 1069.68±0.00 | 1363.36±3.76 | 1165.16±6.11 |
| 54 | Megastigmatrienone | 371.18±4.95 | 3821.29±254.85 | 188.91±58.74 | 4436.51±22.89 |
| 55 | 2-Decanone, 5,9-dimethyl- | 0 | 0 | 0 | 0 |
| 56 | Nonanal | 0 | 159.66±56.54 | 524.59±8.09 | 317.68±48.00 |
| 57 | 3-Decen-5-one | 0 | 0 | 0 | 43.36±0.23 |
| 58 | 2-Butanone, 4-(2,2-dimethyl-6-methylenecyclohexyl)- | 121.72±11.67 | 94.72±0.00 | 49.60±2.23 | 0 |
| 59 | 4-Octanone | 0 | 0 | 0 | 0 |
| 60 | Farnesyl acetone | 376.32±1.16 | 58.06±10.98 | 272.57±1.07 | 267.59±43.50 |
| 61 | 3,5,9-Undecatrien-2-one, 6,10-dimethyl-, (E, E)- | 145.07±3.84 | 0 | 0 | 0 |
| 62 | Bicyclo[3.1.0]hexan-3-one, 4-methyl-1-(1-methylethyl)- | 0 | 0 | 0 | 0 |
| 63 | Undecanal, 2-methyl- | 0 | 0 | 0 | 0 |
| 64 | 3,7-Nonadien-2-one, 4,8-dimethyl- | 0 | 0 | 0 | 0 |
| 65 | 4,8-Dimethylnona-3,8-dien-2-one | 36.30±1.70 | 18.30±5.99 | 141.60±27.91 | 0 |
| 66 | Decanal | 72.57±14.21 | 59.52±17.80 | 123.83±0.15 | 109.19±4.56 |
| 67 | 2-Hexenal, (E)- | 0 | 0 | 0 | 0 |
| 68 | Glutaraldehyde | 0 | 0 | 0 | 0 |
| 69 | Hexanal | 0 | 25.10±0.00 | 60.63±1.11 | 0 |
| 70 | Isophorone | 0 | 15.83±0.00 | 0 | 0 |
| 71 | Benzaldehyde | 80.66±1.10 | 72.36±19.40 | 99.88±24.14 | 354.84±1.86 |
| 72 | 6-Ethyl-5,6-dihydro-2H-pyran-2-one | 0 | 0 | 0 | 279.32±6.10 |
| 73 | 2-Buten-1-one, 1-(2,6,6-trimethyl-1-cyclohexen-1-yl)- | 0 | 0 | 0 | 0 |
| 74 | 4-Hepten-3-one, 5-methyl- | 0 | 0 | 0 | 0 |
| 75 | 3,7-Nonadien-2-one, 4,8-dimethyl- | 0 | 0 | 0 | 0 |
| 76 | 2,6,6-Trimethyl-2-cyclohexene-1,4-dione | 0 | 0 | 0 | 0 |
| 77 | Acetophenone | 29.10±8.44 | 17.26±0.00 | 0 | 38.23±0.20 |
| 78 | Dehydromevalonic lactone | 0 | 0 | 0 | 0 |
| 79 | 2-Nonenal, (E)- | 0 | 0 | 0 | 0 |
| 80 | Butanal, 2-ethyl- | 0 | 0 | 0 | 0 |
| 81 | Dehydromevalonic lactone | 0 | 0 | 0 | 0 |
| 82 | trans-beta-Ionone | 0 | 0 | 0 | 0 |
| 83 | 3-Buten-2-one, 4-(2,2,6-trimethyl-7-oxabicyclo [4.1.0] hept-1-yl)- | 0 | 0 | 0 | 0 |
| 84 | Benzeneacetaldehyde | 0 | 34.65±6.65 | 0 | 113.55±2.93 |
| 85 | 1,3-Cyclohexanedione, 2-(2-propenyl)- | 0 | 0 | 0 | 0 |
| 86 | Solavetivone | 504.01±13.36 | 0 | 0 | 0 |
| 87 | 4,4-Dimethyl-cyclohex-2-en-1-ol | 0 | 0 | 0 | 0 |
| 88 | 7-Oxabicyclo [4.1.0] heptan-2-one, 4,4,6-trimethyl- | 0 | 0 | 0 | 0 |
| 89 | 2-Buten-1-one, 1-(2,6,6-trimethyl-1,3-cyclohexadien-1-yl)- | 0 | 0 | 0 | 0 |
| 90 | 6-Methyl-3,5-heptadiene-2-one | 136.48±100.51 | 59.65±3.01 | 128.94±2.36 | 53.22±23.06 |
| 91 | 2,6,10-Dodecatrienal, 3,7,11-trimethyl-, (Z, E)- | 0 | 0 | 0 | 0 |
| 92 | 2-Piperidinone | 0 | 0 | 0 | 170.87±0.90 |
| 93 | Pentanal | 0 | 0 | 0 | 0 |
| 94 | Dodecanal | 20.89±0.55 | 0 | 0 | 0 |
| 95 | 2-Hexenal | 20.62±0.55 | 0 | 0 | 0 |
| 96 | 1,4-Cyclohexanedione, 2,2,6-trimethyl- | 0 | 13.03±0.00 | 0 | 0 |
| 97 | Hexadecanal | 0 | 0 | 0 | 201.31±1.06 |
|  | **Total** | **5747.94±281.8** | **11034.04±430.5** | **4153.03±135.51** | **9533.92±342.68** |
|  | **Alkane (34)** |  |  |  |  |
| 98 | Decane, 1-(ethenyloxy)- | 109.76±2.91 | 165.99±17.58 | 0 | 0 |
| 99 | Hexadecane | 510.68±20.03 | 481.57±14.45 | 132.38±5.59 | 0 |
| 100 | Octane, 2-chloro- | 0 | 0 | 0 | 0 |
| 101 | Tetradecane | 48.52±9.16 | 131.19±0.00 | 0 | 0 |
| 102 | Dodecane, 2,6,10-trimethyl- | 0 | 0 | 0 | 0 |
| 103 | Pentadecane, 2,6,10-trimethyl- | 0 | 0 | 0 | 0 |
| 104 | Hexadecane, 2,6,10,14-tetramethyl- | 0 | 0 | 0 | 0 |
| 105 | Dodecane, 4,6-dimethyl- | 0 | 0 | 0 | 0 |
| 106 | Octadecane | 34.22±5.04 | 0 | 0 | 0 |
| 107 | Oxirane, dodecyl- | 0 | 0 | 0 | 0 |
| 108 | Undecane, 2-methyl- | 0 | 0 | 0 | 0 |
| 109 | Dodecane | 110.18±3.36 | 24.30±11.67 | 135.54±3.85 | 41.62±0.22 |
| 110 | Eicosane, 2-methyl- | 0 | 0 | 0 | 0 |
| 111 | Decane, 3,7-dimethyl- | 0 | 0 | 0 | 0 |
| 112 | Nonadecane | 0 | 0 | 0 | 0 |
| 113 | Hexadecane, 2-methyl- | 30.70±1.60 | 0 | 0 | 0 |
| 114 | Heptadecane, 2,6-dimethyl- | 0 | 0 | 0 | 0 |
| 115 | Hexadecane, 3-methyl- | 0 | 0 | 0 | 0 |
| 116 | Decane, 5,6-dimethyl- | 0 | 0 | 0 | 0 |
| 117 | Hexadecane, 4-methyl- | 0 | 0 | 0 | 0 |
| 118 | 2-Methyltetracosane | 151.82±10.76 | 0 | 0 | 57.91±0.30 |
| 119 | Heptadecane | 0 | 0 | 0 | 0 |
| 120 | Tridecane, 3-ethyl- | 0 | 0 | 0 | 0 |
| 121 | Heptadecane, 3-methyl- | 0 | 0 | 0 | 0 |
| 122 | Octane, 4,5-dipropyl- | 0 | 0 | 0 | 0 |
| 123 | Tetradecane, 4-ethyl- | 0 | 0 | 0 | 0 |
| 124 | Undecane, 2,5-dimethyl- | 0 | 0 | 0 | 0 |
| 125 | Decane, 2,3,5,8-tetramethyl- | 0 | 0 | 0 | 0 |
| 126 | Decane | 45.52±5.28 | 48.51±0.64 | 0 | 0 |
| 127 | Tridecane | 0 | 0 | 200.36±3.67 | 89.41±0.47 |
| 128 | Dodecane, 1-cyclopentyl-4-(3-cyclopentylpropyl)- | 0 | 0 | 0 | 0 |
| 129 | Cyclohexane, 1-propenyl- | 0 | 0 | 0 | 0 |
| 130 | Tetradecane, 4-methyl- | 0 | 0 | 0 | 0 |
| 131 | Cyclohexane, methyl- | 0 | 0 | 0 | 0 |
|  | **Total** | **1041.4±58.14** | **851.56±44.34** | **468.28±13.11** | **188.94±0.99** |
|  | **Alcohols (17)** |  |  |  |  |
| 132 | 1,2-Dihydrolinalool | 0 | 0 | 0 | 0 |
| 133 | 3,7,11,15-Tetramethyl-2-hexadecen-1-ol | 0 | 0 | 0 | 0 |
| 134 | 1-Dodecanol, 3,7,11-trimethyl- | 325.28±8.62 | 125.98±10.15 | 153.73±12.03 | 217.00±25.69 |
| 135 | Cyclohexanol, 2-methyl-5-(1-methylethenyl)- | 0 | 0 | 0 | 213.28±1.12 |
| 136 | 1-Dodecanol | 0 | 0 | 0 | 0 |
| 137 | 1-Hexanol, 5-methyl-2-(1-methylethyl)- | 0 | 0 | 0 | 0 |
| 138 | 1H-Cycloprop[e]azulen-4-ol, decahydro-1,1,4,7-tetramethyl-, [1aR-(1a. alpha.,4. beta.,4a. beta.,7. alpha.,7a. beta.,7b. alpha.)]- | 38.65±1.02 | 0 | 0 | 0 |
| 139 | trans-2-Dodecen-1-ol | 0 | 0 | 0 | 0 |
| 140 | 1-Hexadecanol | 0 | 0 | 0 | 0 |
| 141 | Phytol | 0 | 0 | 134.85±15.41 | 0 |
| 142 | 1-Tetradecanol | 0 | 0 | 0 | 0 |
| 143 | n-Tridecan-1-ol | 0 | 0 | 0 | 0 |
| 144 | 3-Tetradecyn-1-ol | 0 | 58.83±0.00 | 0 | 0 |
| 145 | Carveol | 0 | 0 | 0 | 0 |
| 146 | n-Nonadecanol-1 | 0 | 0 | 0 | 0 |
| 147 | 1-Octanol, 2,7-dimethyl- | 0 | 0 | 40.07±0.73 | 0 |
| 148 | 2,6,11-Tridecatrien-10-ol, 2,6,10-trimethyl- | 0 | 0 | 0 | 0 |
|  | **Total** | **363.93 ±9.64** | **184.81±10.15** | **328.65±28.17** | **430.28±26.81** |
|  | **Heterocyclic compounds (21)** |  |  |  |  |
| 149 | 2,6-Di-tert-butyl-p-benzoquinone | 0 | 0 | 0 | 0 |
| 150 | n-Hexadecanoic acid | 0 | 0 | 0 | 0 |
| 151 | Anthracene, tetradecahydro- | 0 | 0 | 0 | 0 |
| 152 | Benzene, 1,3,5-tri-tert-butyl- | 0 | 0 | 0 | 0 |
| 153 | 3-(4,8,12-Trimethyltridecyl) furan | 255.11±47.49 | 274.37±7.93 | 119.61±14.50 | 170.94±3.26 |
| 154 | Nonanoic acid | 0 | 0 | 0 | 0 |
| 155 | 5H-1-Pyrindine | 0 | 0 | 0 | 0 |
| 156 | Butane, 1-propoxy- | 0 | 0 | 0 | 0 |
| 157 | Hexadecanedinitrile | 0 | 0 | 0 | 0 |
| 158 | Benzene, (ethoxymethyl)- | 0 | 0 | 0 | 0 |
| 159 | Butyl triacontyl ether | 0 | 0 | 0 | 0 |
| 160 | Naphthalene, 2,3,6-trimethyl- | 0 | 0 | 0 | 0 |
| 161 | 2-Hydroxyhexadecanoic acid | 0 | 0 | 0 | 0 |
| 162 | Azulene | 23.39±0.62 | 22.27±11.70 | 0 | 0 |
| 163 | 1,4,10,13-tetraoxa-7,16-dithiacyclooctadecane | 0 | 0 | 0 | 0 |
| 164 | Benzenamine, N-(1-methylethyl)- | 28.02±0.74 | 39.93±7.31 | 26.36±0.48 | 26.88±2.36 |
| 165 | Ethosuximide | 82.08±6.10 | 52.55±30.01 | 0 | 98.05±18.37 |
| 166 | 2-n-Butyl furan | 0 | 0 | 0 | 0 |
| 167 | Ethosuximide | 82.08±6.10 | 52.55±30.01 | 0 | 98.05±18.37 |
| 168 | Furan, 2-pentyl- | 0 | 0 | 0 | 0 |
| 169 | 3-methylpentanoic acid | 16.47±0.06 | 0 | 0 | 0 |
|  | **Total** | **515.54±61.41** | **441.67±86.96** | **176.17±15.53** | **393.92±42.36** |
