# Supplementary figures and images for: Phyllosphere microbial community of cigar tobacco and its corresponding metabolites
Source: Front Microbiol. 2022 Nov 11;13:1025881. doi: 10.3389/fmicb.2022.1025881 (PMC9691965; doi:10.3389/fmicb.2022.1025881)

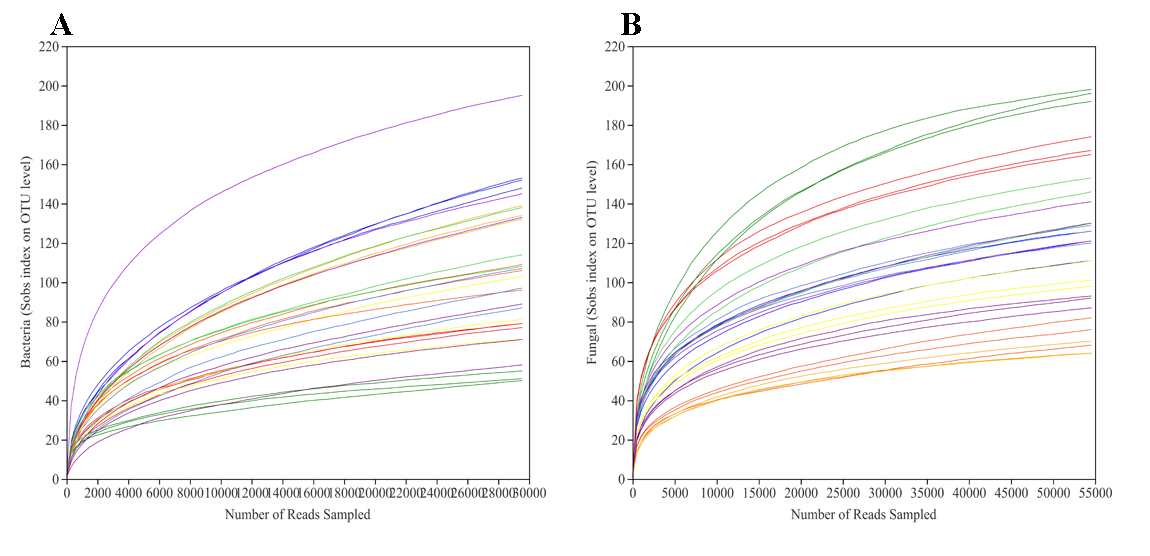

Supplement: Supplementary file 2 [file Image_1.TIF]
